# Supplementary material for: Origin and Correlates of Viral Rebound in SIV-Infected Rhesus Macaques Following ART Discontinuation
Source: bioRxiv. 2025 Aug 30:2025.08.30.673277. Preprint. [Version 1] doi: 10.1101/2025.08.30.673277 (PMC12407973; doi:10.1101/2025.08.30.673277)
Supplement: 1 [file NIHPP2025.08.30.673277V1-supplement-1.pdf]

bioRxiv preprint doi: https://doi.org/10.1101/2025.08.30.673277; this version posted September 1, 2025. The copyright holder for this preprint (which was not certified by peer review) is the author/funder, who has granted bioRxiv a license to display the preprint in perpetuity. It is made available under aCC-BY 4.0 International license.

Supplementary Figure Legends

Figure S1 | Distribution and quantification of intact proviruses in PBMCs during ART suppression and at necropsy. Quantification of intact proviruses in PBMC using the SIV-specific intact proviral DNA assay (IPDA) at (A) week 36 post-infection, (B) week 66 post-infection, and (C) necropsy. Data is shown for each ART initiation group: day 6 (red diamonds), day 9 (blue circles), and day 12 (green triangles). Black lines indicate mean ± standard deviation. * $p<0.0332$, ** $p<0.0021$. Open symbols are below limit of detection.

Figure S2 | Tissue distribution of cell associated vDNA and vRNA at week 21. Detection of (A) SIV cell-associated DNA (vDNA) and (B) SIV cell-associated RNA (vRNA) in various tissues at week 21 following infection, including in bronchoalveolar lavage (BAL) cells, colon, left axillary LN, mesenteric LN, liver, bone marrow, spleen, and small intestine biopsies. Data is shown for each ART initiation group: day 6 (red diamonds), day 9 (blue circles), and day 12 (green triangles). Black lines indicate mean ± standard deviation. Unfilled circles represent samples in which no replicates were positive for vDNA or vRNA, and an imputed threshold value was plotted to indicate levels below the detection limit.

Figure S3 | SIV-specific humoral and cellular immune responses during ART and after ART discontinuation. The median titers of serum antibodies to the SIVmac239 gp160 recombinant protein are shown for all treatment groups, day 6 (red), day 9 (blue), and day 12 (green) (A) during ART suppression and (B) after treatment interruption. ELISA antibody titers are shown at (C) week 66 post-infection and (D) day 12 (at necropsy) after treatment interruption. PBMC IFN-γ ELISPOT

bioRxiv preprint doi: https://doi.org/10.1101/2025.08.30.673277; this version posted September 1, 2025. The copyright holder for this preprint (which was not certified by peer review) is the author/funder, who has granted bioRxiv a license to display the preprint in perpetuity. It is made available under aCC-BY 4.0 International license.

assays using pools of peptides spanning the SIVmac239 Env, Gag, and Pol proteins at (E) week 33 post-infection and (F) week 66 post-infection, with the dotted line indicating the limit of detection. (G) PBMC Gag-specific cellular immune responses at week 66 following infection. (H) SIV-specific IFN-γ + CD4 T cells in spleen at necropsy and (I) SIV-specific TNF-α + CD4 T cells in spleen at necropsy. Black lines indicate mean ± standard deviation. * p<0.0332, ** p<0.0021.

Figure S4 | Dynamics of rebound after ART discontinuation. Rebound plasma viral load (RNA copies/mL) from time of ART discontinuation to necropsy for each animal, grouped by ART initiation time. The emergence and proportional contribution to total viral load of distinct barcodes is shown longitudinally, with heatmap color coding of barcodes corresponding to when they were first detected in rebound plasma. For all viremic animals, the number of newly identified barcode lineages per day is shown. The time to plasma viral load rebound of >50 copies/mL is labeled in red as the number of days following ART discontinuation.

Figure S5 | Rebounding barcodes labeled in barcode distribution of plasma viremia during primary infection prior to ART initiation. The barcode plasma viral loads ($\log_{10}$ RNA copies/mL) prior to ART initiation is shown for each animal, grouped by ART initiation time. Grey dots correspond to barcodes that were not detected in rebound plasma, whereas colored diamonds indicate rebounding lineages (n=175).

Figure S6 | Total barcode vRNA and vDNA levels in necropsy tissues vs. pre-ART and rebound plasma viremia. For animals starting ART at day 6 (left), 9 (middle) and 12 (right), the primary infection viremia prior to ART initation ($\log_{10}$, x-axis) for each individual identified

bioRxiv preprint doi: https://doi.org/10.1101/2025.08.30.673277; this version posted September 1, 2025. The copyright holder for this preprint (which was not certified by peer review) is the author/funder, who has granted bioRxiv a license to display the preprint in perpetuity. It is made available under aCC-BY 4.0 International license.

barcode is compared against total vRNA (top) and vDNA (bottom) ($\log_{10}$, y-axis) for that barcode across all analyzed necropsy tissues. Colored dots indicate barcode lineages identified in rebound viremia, with the color scale indicating the level of each individual barcode in rebound viremia ($\log_{10}$) at necropsy. Grey dots indicate barcodes that were not detected in rebound plasma viremia. Overall, higher levels of individual rebounding vRNA barcodes in tissues (color) were associated with higher levels in peak primary infection viremia and higher representation in rebound viremia.

Figure S7 | Viral barcode clonotypes in necropsy tissues from a non-rebounding RM TP5 and a single-barcode rebounder RM L970. (A) Plasma viral load dynamics following ART discontinuation for RMs TP5 (top) and L970 (bottom); red lines indicate individual viral load trajectories. RM TP5 exhibited no viral rebound through the 12-day monitoring period. (B) Proportional distributions of viral barcode clonotypes in plasma viremia during primary infection prior to ART initiation for RMs TP5 (top) and L970 (bottom); for L970, a single plasma barcode (BC.640), first detectable at day 11 following ART discontinuation is highlighted in red (bottom left panel). The bottom right panel shows calculated rebound viral growth curves for each rebounding barcode lineage with estimated time to a single copy in rebound viremia indicated. Red line indicates the dominant rebounding lineage. (C) Necropsy tissue distribution of vRNA and vDNA from primary infection for each animal. For RM L970, technical issues precluded vRNA barcode analysis in tissues, and thus vRNA values represent total vRNA rather than barcode vRNA. Grouped tissue categories are: GI tract (blue), GI tract draining lymph nodes (red), non-GI lymph tissues (green), non-lymphoid tissue (purple).

bioRxiv preprint doi: https://doi.org/10.1101/2025.08.30.673277; this version posted September 1, 2025. The copyright holder for this preprint (which was not certified by peer review) is the author/funder, who has granted bioRxiv a license to display the preprint in perpetuity. It is made available under aCC-BY 4.0 International license.

Figure S8 | Viral barcode clonotypes in necropsy tissues and contributions to rebound viremia – RM L991, 2 rebounding barcodes. (A) Plasma viral load dynamics following ART discontinuation; red line indicates RM L991. (B) Left panel: Proportional distribution of viral barcode clonotypes in plasma viremia during primary infection prior to ART initiation, with the two barcodes found in rebound plasma at necropsy (BC.897 and BC.2604) highlighted. Middle panel: The calculated plasma viral load values for the two barcodes identified in the single day 12 timepoint, BC.897 and BC.2604, are shown. (C) Necropsy tissue distribution of vRNA and vDNA for the two barcodes identified in necropsy rebound plasma. Grouped tissue categories are: GI tract (blue), GI tract draining lymph nodes (red), non-GI lymph tissues (green), non-lymphoid tissue (purple). (D) Necropsy tissue vRNA levels for the two barcodes documented in rebound plasma viremia at necropsy; outlier analysis did not allow identification of presumptive tissue origin sites for either.

Figure S9 | Viral barcode clonotypes in necropsy tissues and contributions to rebound viremia – RM L677, 5 rebounding barcodes. (A) Plasma viral load dynamics following ART discontinuation; red line indicates the viral load trajectory of RM L991. (B) Left panel: Proportional distribution of viral barcode clonotypes in plasma viremia during primary infection prior to ART initiation, with the 5 barcodes found in day 12 necropsy rebound plasma (BC.2068, BC.2, BC.5111, BC.001, and BC.80) highlighted. Middle panel: Calculated levels of plasma vRNA at necropsy for the 5 rebounding barcode lineage with estimated time to a single copy in rebound viremia indicated. Red line indicates the dominant rebounding lineage. Grey lines correspond to clones detected in rebound plasma but without an identified tissue origin site. Right panel: Proportional distribution of rebound viral barcode clonotypes in necropsy plasma at

bioRxiv preprint doi: https://doi.org/10.1101/2025.08.30.673277; this version posted September 1, 2025. The copyright holder for this preprint (which was not certified by peer review) is the author/funder, who has granted bioRxiv a license to display the preprint in perpetuity. It is made available under aCC-BY 4.0 International license.

necropsy with barcodes for which a origin site was identified highlighted. (C) vRNA and vDNA distribution of the 5 rebounding barcodes (color-coded to panel B) in necropsy tissues. Grouped tissue categories are: GI tract (blue), GI tract draining lymph nodes (red), non-GI lymph tissues (green), non-lymphoid tissue (purple). Colored upward-facing triangles represent the 2 rebounding barcodes with tissue origin sites (BC.2 and BC.80) identified by outlier analysis indicated by the large, filled symbols. Unfilled symbols indicate barcodes for which outlier analysis did not identify a presumptive tissue origin site. (D) Viral barcode SIV RNA copies in tissue, grouped by barcode ID. Each point represents an individual barcode detected in necropsy tissue. Large, filled triangles indicate tissue barcode vRNA levels in tissue origin sites.

Figure S10 | Viral barcode clonotypes in necropsy tissues and contributions to rebound viremia – RM DHJG, 6 rebounding barcodes. (A) Plasma viral load dynamics following ART discontinuation; red line indicates RM DHJG. (B) Left panel: Proportional distribution of viral barcode clonotypes in plasma viremia during primary infection prior to ART initiation, with barcodes found in rebound plasma highlighted. Middle panel: Calculated rebound viral growth curves for each rebounding barcode lineage with estimated time to a single copy in rebound viremia indicated. Red line indicates the dominant rebounding lineage (BC.3489). Grey lines correspond to barcodes detected in rebound plasma but without an identified presumptive tissue origin site. Right panel: Proportional distribution of rebound viral barcode clonotypes vRNA in necropsy plasma necropsy with one barcode with a tissue origin site identified by outlier analysis highlighted (large, filled triangle). (C) vRNA and vDNA distribution of all rebounding barcodes (color-coded to panel B) in necropsy tissues. Open symbols indicate barcodes without tissue origin site detected. Colored upward-facing triangles represent the one rebounding barcode with a tissue

bioRxiv preprint doi: https://doi.org/10.1101/2025.08.30.673277; this version posted September 1, 2025. The copyright holder for this preprint (which was not certified by peer review) is the author/funder, who has granted bioRxiv a license to display the preprint in perpetuity. It is made available under aCC-BY 4.0 International license.

origin site in a gastric LN indicated by the large symbol. Grouped tissue categories are: GI tract (blue), GI tract draining lymph nodes (red), non-GI lymph tissues (green), non-lymphoid tissue (purple). (D) Viral barcode vRNA copies in necropsy tissue, grouped by barcode ID. Each point represents an individual barcode detected in tissue. Outlier analysis identified BC.1665 as the sole rebounding barcode for which a tissue origin site could be identified.

Figure S11 | Viral barcode clonotypes in necropsy tissues and contributions to rebound viremia – RM L608, 7 rebounding barcodes. (A) Plasma viral load dynamics following ART discontinuation; red line indicates RM L608. (B) Left panel: Proportional distribution of all viral barcode clonotypes in plasma viremia during primary infection prior to ART initiation, with barcodes found in rebound plasma highlighted. Middle panel: Calculated rebound viral growth curves for each rebounding barcode lineage with estimated time to a single copy in rebound viremia indicated. Red line indicates the dominant rebounding lineage. Grey symbols correspond to barcodes detected in rebound plasma for which no presumptive tissue origin site was identified. Right panel: Proportional distribution of rebound plasma vRNA barcode clonotypes in necropsy plasma with barcodes for which tissue origin sites were identified highlighted. (C) vRNA and vDNA distribution of all rebounding barcodes (color-coded to panel B) in necropsy tissues. Colored upward-facing triangles represent rebounding barcodes with a tissue origin site, indicated by the large, filled triangles. Open symbols indicate barcodes without a presumptive tissue origin site identified. Grouped tissue categories are: GI tract (blue), GI tract draining lymph nodes (red), non-GI lymph tissues (green), non-lymphoid tissue (purple). (D) SIV RNA levels in necropsy tissues for barcodes present in rebound viremia, grouped by barcode ID. Each point represents an

bioRxiv preprint doi: https://doi.org/10.1101/2025.08.30.673277; this version posted September 1, 2025. The copyright holder for this preprint (which was not certified by peer review) is the author/funder, who has granted bioRxiv a license to display the preprint in perpetuity. It is made available under aCC-BY 4.0 International license.

individual barcode detected in necropsy tissue. Tissue origin sites identified by outlier analysis for barcodes BC.5071, BC2.878, and BC.4101 are indicated by large, filled triangles.

Figure S12 | Viral barcode clonotypes in necropsy tissues and contributions to rebound viremia – RM DHGI, 9 rebounding barcodes. (A) Plasma viral load dynamics following ART discontinuation; red line indicates RM DHGI. (B) Left panel: Proportional distribution of viral barcode clonotypes in plasma viremia during primary infection prior to ART initiation, with all barcodes found in rebound plasma highlighted. Middle panel: Calculated rebound viral growth curves of each rebounding barcode lineage with estimated time to a single copy in rebound viremia indicated. Red line indicates the dominant rebounding lineage. Grey lines correspond to clones detected in rebound plasma but without an identified presumptive tissue origin site. Right panel: Proportional distribution of rebound viral barcode clonotypes in necropsy plasma with barcodes for which a tissue origin site was identified highlighted. (C) vRNA and vDNA distribution of all rebounding barcodes (color-coded to panel B) in necropsy tissues. Colored upward-facing triangles represent the one rebounding barcode (BC.1592) for which outlier analysis indicated a tissue origin site in cecum plotted with the by large, filled triangle. Open symbols indicate barcodes without presumptive tissue origin sites detected. Grouped tissue categories are: GI tract (blue), GI tract draining lymph nodes (red), non-GI lymph tissues (green), non-lymphoid tissue (purple). (D) Viral barcode SIV RNA copies in tissue, grouped by barcode ID. Each point represents an individual barcode detected in necropsy tissue, with the tissue origin for BC.1592 plotted as a filled triangle.

bioRxiv preprint doi: https://doi.org/10.1101/2025.08.30.673277; this version posted September 1, 2025. The copyright holder for this preprint (which was not certified by peer review) is the author/funder, who has granted bioRxiv a license to display the preprint in perpetuity. It is made available under aCC-BY 4.0 International license.

Figure S13 | Viral barcode clonotypes in necropsy tissues and contributions to rebound viremia – RM L990, 13 rebounding barcodes. (A) Plasma viral load dynamics following ART discontinuation; red line indicates RM L990. (B) Left panel: Proportional distribution of viral barcode clonotypes in plasma viremia during primary infection prior to ART initiation, with all barcodes found in rebound plasma highlighted. Middle panel: Calculated rebound viral growth curves of each rebounding barcode lineage with estimated time to a single copy in rebound viremia indicated. Red line indicates the dominant rebounding lineage. Grey lineages correspond to barcodes detected in rebound plasma for which a presumptive tissue origin site was not identified. Right panel: Proportional distribution of rebound viral barcode clonotypes in plasma at necropsy with barcodes for which tissue origin sites were identified highlighted. (C) vRNA and vDNA distribution of all rebounding barcodes (color-coded to panel B) in necropsy tissues. Colored upward-facing triangles represent rebounding barcodes with tissue origin site indicated by the large symbol. Open symbols indicate barcodes without tissue origin site detected. Grouped tissue categories are: GI tract (blue), GI tract draining lymph nodes (red), non-GI lymph tissues (green), non-lymphoid tissue (purple). (D) Viral barcode SIV RNA copies in necropsy tissues, grouped by barcode ID. Each point represents an individual barcode detected in a necropsy tissue, with large, filled triangles indicating outliers allowing identification of tissue origin sites for BC.2127, BC.743, BC.377, and BC.5223.

Figure S14 | Viral barcode clonotypes in necropsy tissues and contributions to rebound viremia – RM L956, 14 rebounding barcodes. (A) Plasma viral load dynamics following ART discontinuation; red line indicates RM L956 (B) Left panel: Proportional distribution of viral barcode clonotypes in plasma viremia during primary infection prior to ART initiation, with all

bioRxiv preprint doi: https://doi.org/10.1101/2025.08.30.673277; this version posted September 1, 2025. The copyright holder for this preprint (which was not certified by peer review) is the author/funder, who has granted bioRxiv a license to display the preprint in perpetuity. It is made available under aCC-BY 4.0 International license.

barcodes found in rebound plasma highlighted. Middle panel: Calculated rebound viral growth curves of each rebounding barcode lineage with estimated time to a single copy in rebound viremia indicated. Red line indicates the dominant rebounding lineage. Grey lines correspond to barcodes detected in rebound plasma for which a presumptive tissue origin site was not identified. Right panel: Proportional distribution of rebound viral barcode clonotypes in plasma at necropsy with barcodes having tissue origins highlighted. (C) vRNA and vDNA distribution of all rebounding barcodes (color-coded to panel B) in necropsy tissues. Colored upward-facing triangles represent rebounding barcodes with tissue origin sites indicated by large, filled triangles. Open symbols indicate barcodes without tissue origin site detected. Grouped tissue categories are: GI tract (blue), GI tract draining lymph nodes (red), non-GI lymph tissues (green), non-lymphoid tissue (purple). (D) Viral barcode SIV RNA copies in necropsy tissues, grouped by barcode ID. Each point represents an individual barcode detected in a necropsy tissue, with large, filled triangles indicating outliers allowing identification of tissue origin sites for BC.447, BC.6182, BC.2734, BC.311, BC.3948, and BC.203.

Figure S15 | Viral barcode clonotypes in necropsy tissues and contributions to rebound viremia – RM DHIX, 16 rebounding barcodes. (A) Plasma viral load dynamics following ART discontinuation; red line indicates RM DHIX. (B) Left panel: Proportional distribution of viral barcode clonotypes in plasma viremia during primary infection prior to ART initiation, with all barcodes found in rebound plasma highlighted. Middle panel: Calculated rebound viral growth curves of each rebounding barcode lineage with estimated time to a single copy in rebound viremia indicated. Red line indicates the dominant rebounding lineage. Grey lines correspond to barcodes detected in rebound plasma for which a presumptive tissue origin site was not identified. Right

bioRxiv preprint doi: https://doi.org/10.1101/2025.08.30.673277; this version posted September 1, 2025. The copyright holder for this preprint (which was not certified by peer review) is the author/funder, who has granted bioRxiv a license to display the preprint in perpetuity. It is made available under aCC-BY 4.0 International license.

panel: Proportional distribution of rebound viral barcode clonotypes in necropsy plasma with barcodes having tissue origin sites identified highlighted. (C) vRNA and vDNA distribution of all rebounding barcodes (color-coded to panel B) in necropsy tissues. Colored upward-facing triangles represent rebounding barcodes for which outlier analysis identified a tissue origin site, indicated by large, filled triangles. Open symbols indicate barcodes for which a tissue origin site was not detected. Grouped tissue categories are: GI tract (blue), GI tract draining lymph nodes (red), non-GI lymph tissues (green), non-lymphoid tissue (purple). (D) Viral barcode SIV RNA copies in necropsy tissues, grouped by barcode ID. Each point represents an individual barcode detected in a necropsy tissue. Large, filled triangles indicated tissue specimens identified by outlier analysis as tissue origin sites for barcodes BC.1836, BC.1279, BC.3432, BC.279, BC.2647, BC.2381, BC.6175, and BC.959 identified in necropsy rebound viremia.

Figure S16 | Viral barcode clonotypes in necropsy tissues and contributions to rebound viremia – RM DHEZ, 33 rebounding barcodes. (A) Plasma viral load dynamics following ART discontinuation; red line indicates RM DHEZ. (B) Left panel: Proportional distribution of viral barcode clonotypes in plasma viremia during primary infection prior to ART initiation, with all barcodes found in rebound plasma highlighted. Middle panel: Calculated rebound viral growth curves for each rebounding barcode lineage with estimated time to a single copy in rebound viremia indicated. Red line indicates the dominant rebounding lineage. Grey lines correspond to clones detected in rebound plasma for which no presumptive tissue origin site was identified. Right panel: Proportional distribution of rebound viral barcode clonotypes in necropsy plasma with barcodes for which a tissue origin site was identified highlighted. (C) vRNA and vDNA distribution of all rebounding barcodes (color-coded to panel B) in necropsy tissues. Colored

bioRxiv preprint doi: https://doi.org/10.1101/2025.08.30.673277; this version posted September 1, 2025. The copyright holder for this preprint (which was not certified by peer review) is the author/funder, who has granted bioRxiv a license to display the preprint in perpetuity. It is made available under aCC-BY 4.0 International license.

upward-facing triangles represent rebounding barcodes with tissue origin site indicated by the large symbol. Open symbols indicate barcodes without tissue origin site detected. Grouped tissue categories are: GI tract (blue), GI tract draining lymph nodes (red), non-GI lymph tissues (green), non-lymphoid tissue (purple). (D) Viral barcode SIV RNA copies in necropsy tissues, grouped by barcode ID. Each point represents an individual barcode detected in a necropsy tissue. Large, filled triangles indicated tissue specimens identified by outlier analysis as tissue origin sites for barcodes BC.8377, BC.4530, BC.2271, BC.2279, BC.428, BC.4678, BC.2239, BC.1193, and BC.7491 identified in necropsy rebound viremia.

Figure S17 | Viral barcode clonotypes in necropsy tissues and contributions to rebound viremia – RM L997, 38 rebounding barcodes. (A) Plasma viral load dynamics following ART discontinuation; red line indicates RM L997. (B) Left panel: Proportional distribution of viral barcode clonotypes in plasma viremia during primary infection prior to ART initiation, with all barcodes found in rebound plasma highlighted. Middle panel: Calculated rebound viral growth curves for each rebounding barcode lineage with estimated time to a single copy in rebound viremia indicated. Red line indicates the dominant rebounding lineage. Grey lines correspond to clones detected in rebound plasma for which no presumptive tissue origin site was identified. Right panel: Proportional distribution of rebound viral barcode clonotypes in necropsy plasma with barcodes for which a tissue origin site was identified highlighted. (C) vRNA and vDNA distribution of all rebounding barcodes (color-coded to panel B) in necropsy tissues. Colored upward-facing triangles represent rebounding barcodes with tissue origin site indicated by the large symbol. Open symbols indicate barcodes without tissue origin site detected. Grouped tissue categories are: GI tract (blue), GI tract draining lymph nodes (red), non-GI lymph tissues (green),

bioRxiv preprint doi: https://doi.org/10.1101/2025.08.30.673277; this version posted September 1, 2025. The copyright holder for this preprint (which was not certified by peer review) is the author/funder, who has granted bioRxiv a license to display the preprint in perpetuity. It is made available under aCC-BY 4.0 International license.

non-lymphoid tissue (purple). (D) Viral barcode SIV RNA copies in necropsy tissues, grouped by barcode ID. Each point represents an individual barcode detected in a necropsy tissues, with large, filled triangles indicating tissue specimens identified in outlier analysis as tissue origins for barcodes contributing to rebound viremia, including BC.57, BC.314, BC.424, BC.2774, BC.887, BC.2125, BC.696, BC.3717, BC.2978, and BC.1120.

Figure S18 | Principal component analysis of transcriptomic and proteomic data. Colors represent different timepoints following ART discontinuation relative to the day of rebound, defined for the purpose of this analysis as viral load >50 copies/mL.

Figure S19 | Genes driving transcriptomic upregulation in pathways at pre-rebound timepoints relative to baseline following ART discontinuation. Genes contributing to transcriptomic pathway upregulation in pre-rebound samples (viral load <50 cp/mL) compared to baseline (last timepoint prior to ATI) following ART discontinuation. (A) Genes driving upregulation of immune-related pathways and (B) genes associated with metabolism, cell cycle, and chromatin-remodeling pathways.

Figure S20 | Correlation of transcriptomic pathways with time to viral rebound. (A) Spearman correlation analysis of transcriptomic pathways with time to viral rebound following ART discontinuation. Pathways with significant correlations ($p<0.01$) are shown, with red indicating positive correlations and blue indicating negative correlations. (B) Scatterplots of transcriptomic pathways correlated with time to viral rebound. The x-axis shows the SLEA Z-score at day 0, and the y-axis shows the time to viral rebound. Spearman correlations and *p*-values are

bioRxiv preprint doi: https://doi.org/10.1101/2025.08.30.673277; this version posted September 1, 2025. The copyright holder for this preprint (which was not certified by peer review) is the author/funder, who has granted bioRxiv a license to display the preprint in perpetuity. It is made available under aCC-BY 4.0 International license.

shown for each pathway. (C) Univariate Cox proportional hazards model identifies pathways associated with shorter or longer times to viral rebound, displaying the beta coefficient and 95% confidence intervals. Red denotes pathways linked to shorter rebound times, while blue represents pathways associated with longer rebound times. Color gradient intensity reflects the significant level as Wald $p<0.01$.

Figure S21 | Proteomic changes following ART discontinuation before and after viral rebound relative to baseline. Upregulated proteomic pathways compared to baseline (last timepoint prior to ATI) are shown for pre-rebound samples (plasma viral loads <50 cp/mL) in (A) immune-related pathways and (B) metabolism, cell cycle, and chromatin remodeling pathways. Time course analysis depicts upregulated pathways relative to baseline for (C) immune pathways and (D) metabolism, cell cycle, and chromatin remodeling pathways across specific timepoints: baseline, days -9 to -4, days -3 to -1, and on the day of rebound and post-rebound >50 copies/mL. Each day is relative to the first timepoint with plasma viral load >50 copies/mL. Circle size reflects significance, while the color gradient represents the overlap ratio.

bioRxiv preprint doi: https://doi.org/10.1101/2025.08.30.673277; this version posted September 1, 2025. The copyright holder for this preprint (which was not certified by peer review) is the author/funder, who has granted bioRxiv a license to display the preprint in perpetuity. It is made available under aCC-BY 4.0 International license.

Supplementary Table Legends

Table S1 | Reactivation rates and viral dynamics across ART groups. Reactivation rates and viral rebound characteristics for SIV-infected macaques following ART discontinuation, organized by ART initiation group (day 6, day 9, day 12). Plasma viral load at necropsy (SIV copies/mL), rebound growth rate (log_{10} RNA/day), number of barcodes detected in plasma following ART discontinuation, and estimated reactivation rate (events/day) are reported for each animal. "N/A" indicates values not applicable for two animals (TP5 and DHHN) that did not rebound by the definition of viremia >50 copies/mL by day 12 following ART discontinuation.

Table S2 | RM demographics and tissues. Clinical data and the number of tissues collected per animal at necropsy.

Table S3 | Linear regression and logistic regression models assessing virologic and tissue correlates of viral rebound. These tables summarize statistical models evaluating predictors of viral rebound following ART discontinuation in SIV-infected RMs. (A) Effect of barcode plasma viral loads prior to ART initiation on the probability of rebound using logistic regression. (B) Linear regression models relating total tissue-associated vDNA and vRNA levels (log_{10}) to rebound barcode plasma viral loads. (C) Effect of anatomical tissue group (GI tract, GI-draining LNs, and non-GI LNs) on the probability of rebound using logistic regression. Odds ratios, 95% confidence intervals, and *p*-values are reported. Model fit parameters including AICc, variance components, and R^2 values are included where applicable.

bioRxiv preprint doi: https://doi.org/10.1101/2025.08.30.673277; this version posted September 1, 2025. The copyright holder for this preprint (which was not certified by peer review) is the author/funder, who has granted bioRxiv a license to display the preprint in perpetuity. It is made available under aCC-BY 4.0 International license.

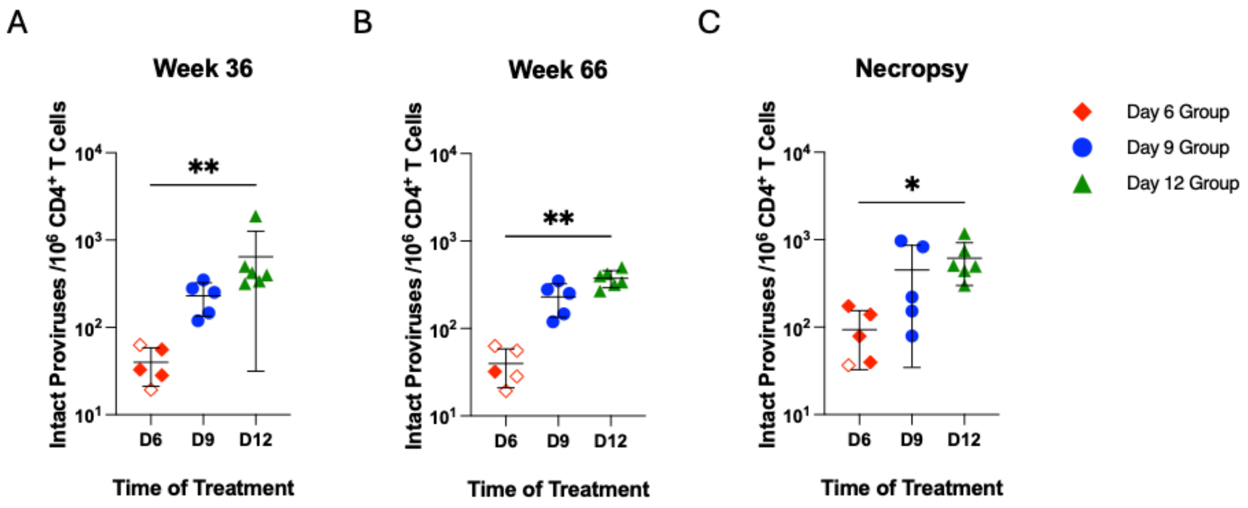

Figure S1.

bioRxiv preprint doi: https://doi.org/10.1101/2025.08.30.673277; this version posted September 1, 2025. The copyright holder for this preprint (which was not certified by peer review) is the author/funder, who has granted bioRxiv a license to display the preprint in perpetuity. It is made available under aCC-BY 4.0 International license.

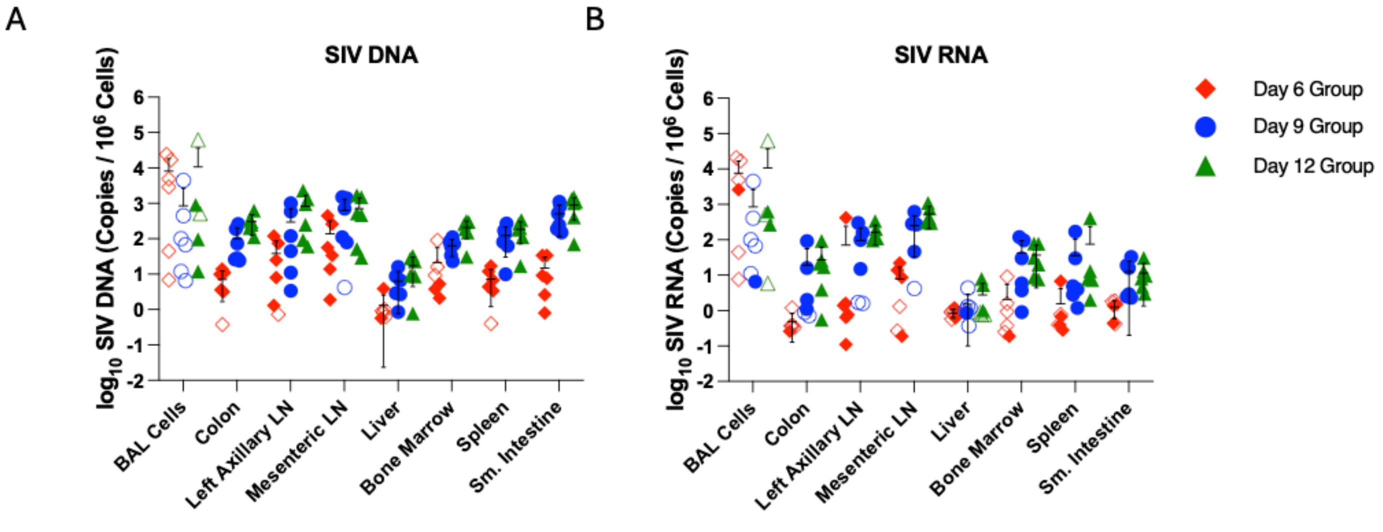

Figure S2.

bioRxiv preprint doi: https://doi.org/10.1101/2025.08.30.673277; this version posted September 1, 2025. The copyright holder for this preprint (which was not certified by peer review) is the author/funder, who has granted bioRxiv a license to display the preprint in perpetuity. It is made available under aCC-BY 4.0 International license.

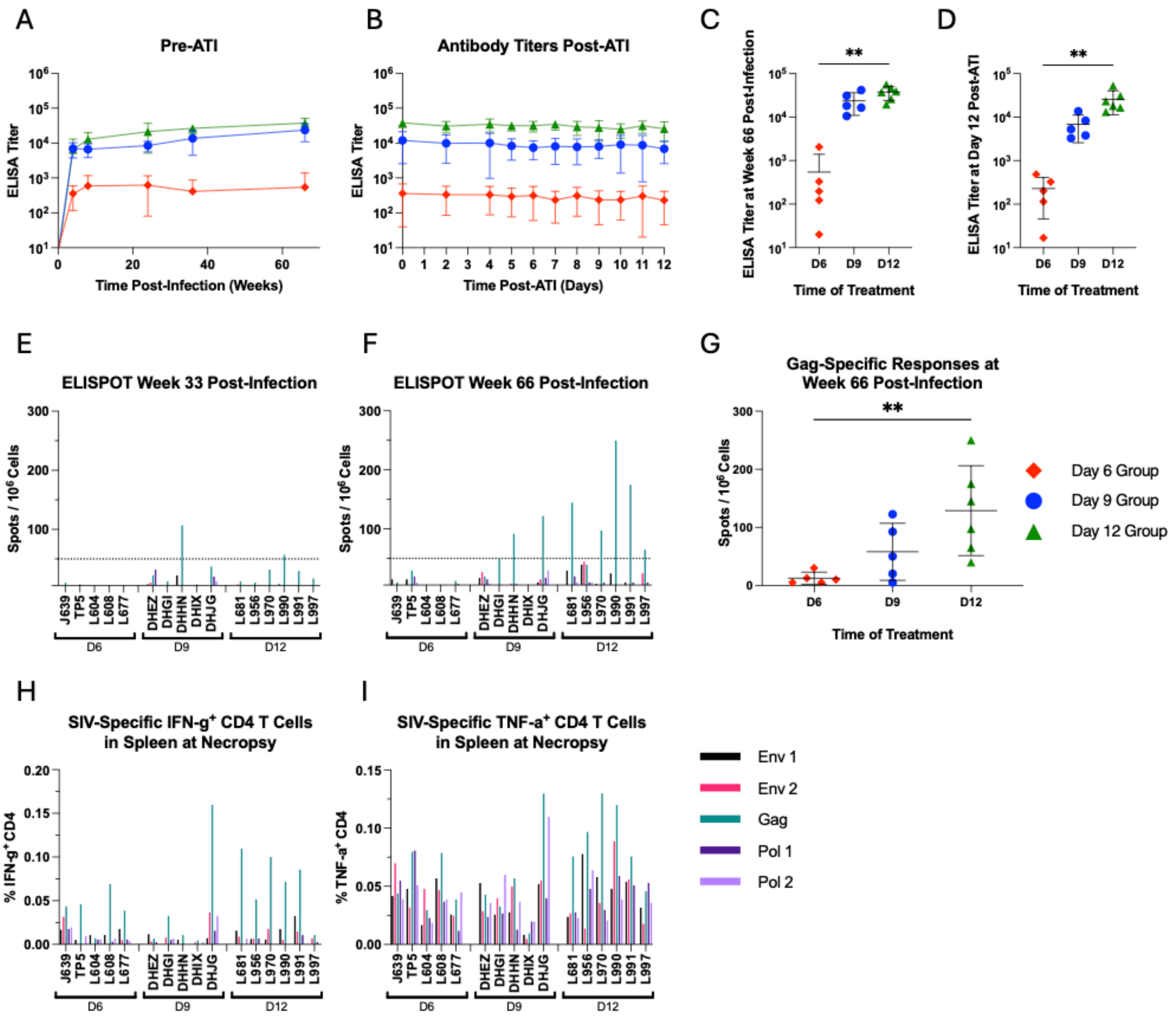

Figure S3.

bioRxiv preprint doi: https://doi.org/10.1101/2025.08.30.673277; this version posted September 1, 2025. The copyright holder for this preprint (which was not certified by peer review) is the author/funder, who has granted bioRxiv a license to display the preprint in perpetuity. It is made available under aCC-BY 4.0 International license.

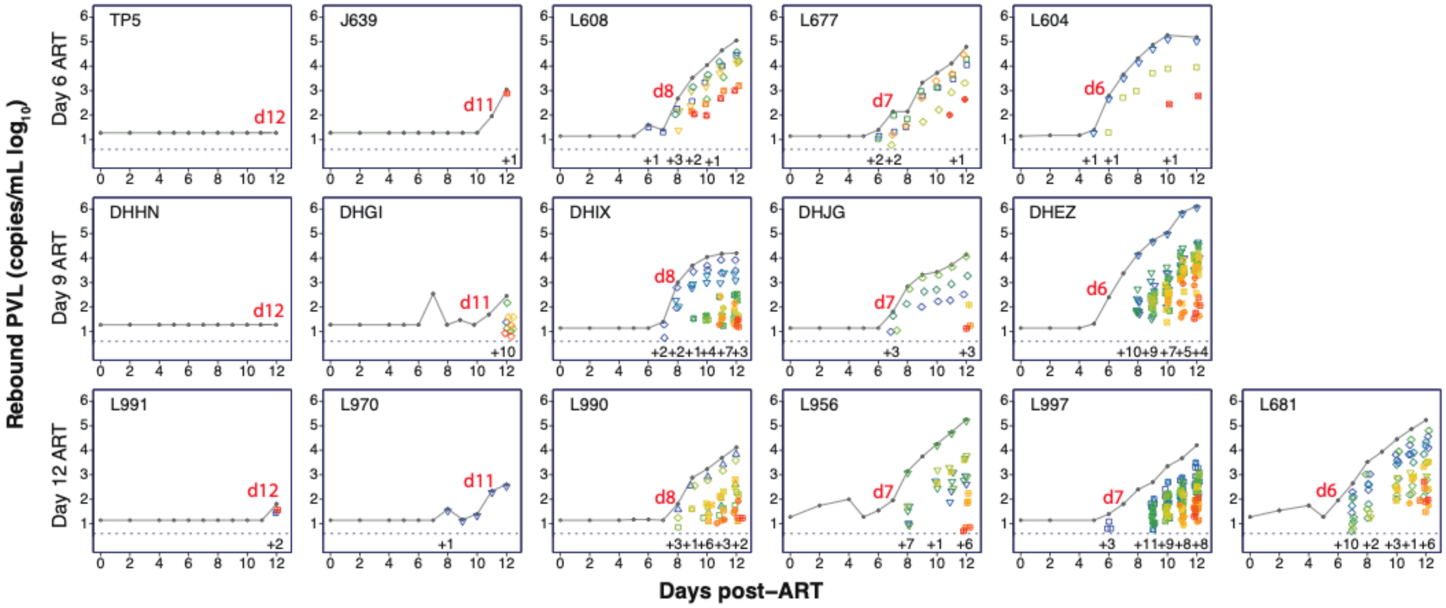

Figure S4.

bioRxiv preprint doi: https://doi.org/10.1101/2025.08.30.673277; this version posted September 1, 2025. The copyright holder for this preprint (which was not certified by peer review) is the author/funder, who has granted bioRxiv a license to display the preprint in perpetuity. It is made available under aCC-BY 4.0 International license.

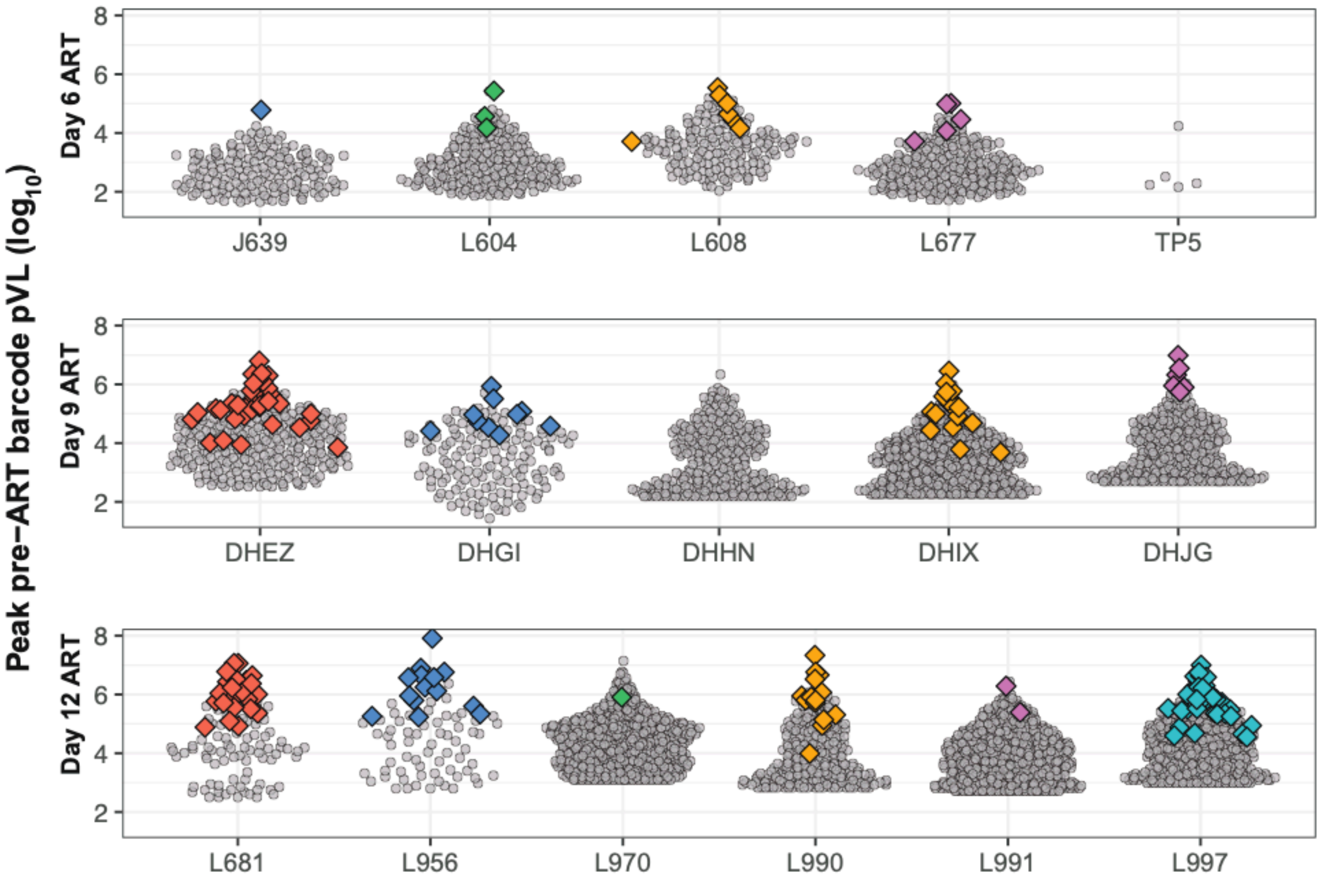

Figure S5.

bioRxiv preprint doi: https://doi.org/10.1101/2025.08.30.673277; this version posted September 1, 2025. The copyright holder for this preprint (which was not certified by peer review) is the author/funder, who has granted bioRxiv a license to display the preprint in perpetuity. It is made available under aCC-BY 4.0 International license.

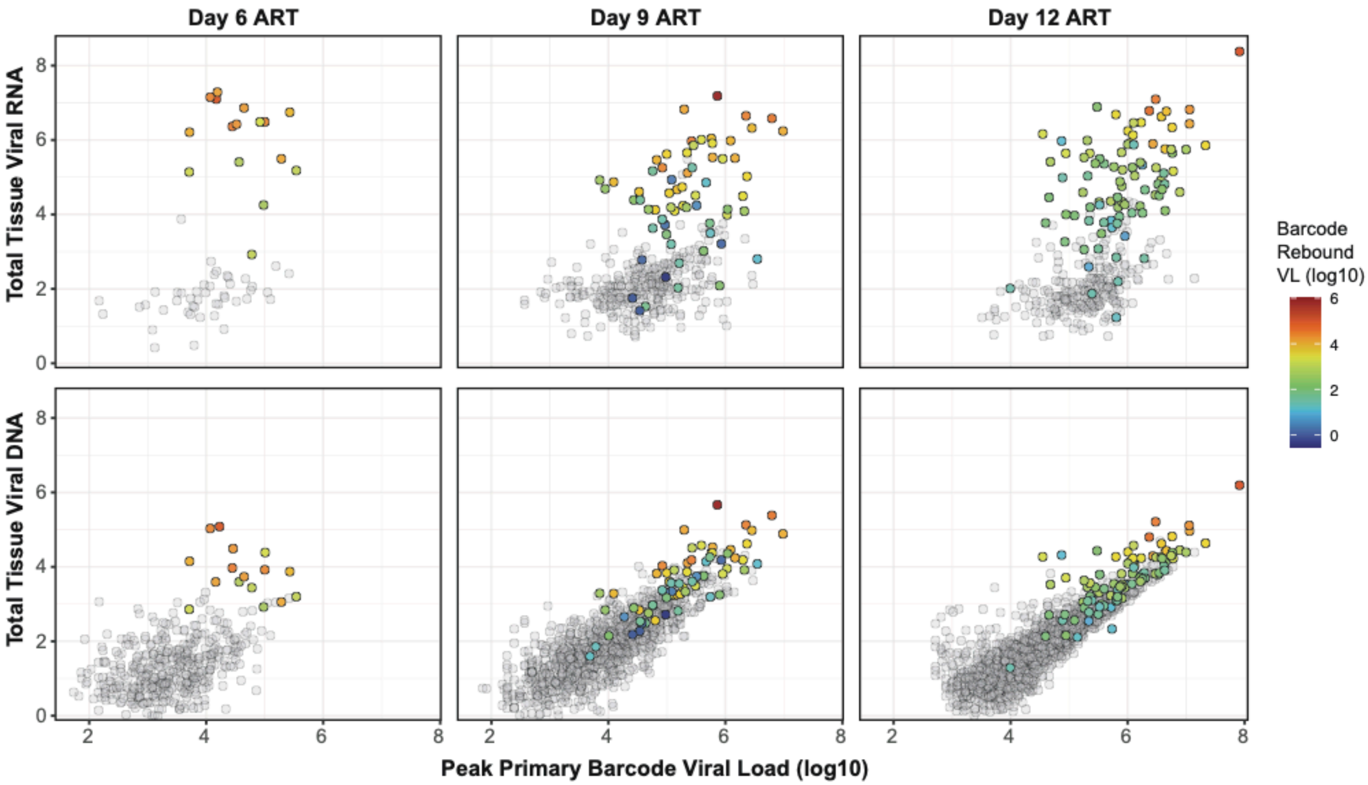

Figure S6.

bioRxiv preprint doi: https://doi.org/10.1101/2025.08.30.673277; this version posted September 1, 2025. The copyright holder for this preprint (which was not certified by peer review) is the author/funder, who has granted bioRxiv a license to display the preprint in perpetuity. It is made available under aCC-BY 4.0 International license.

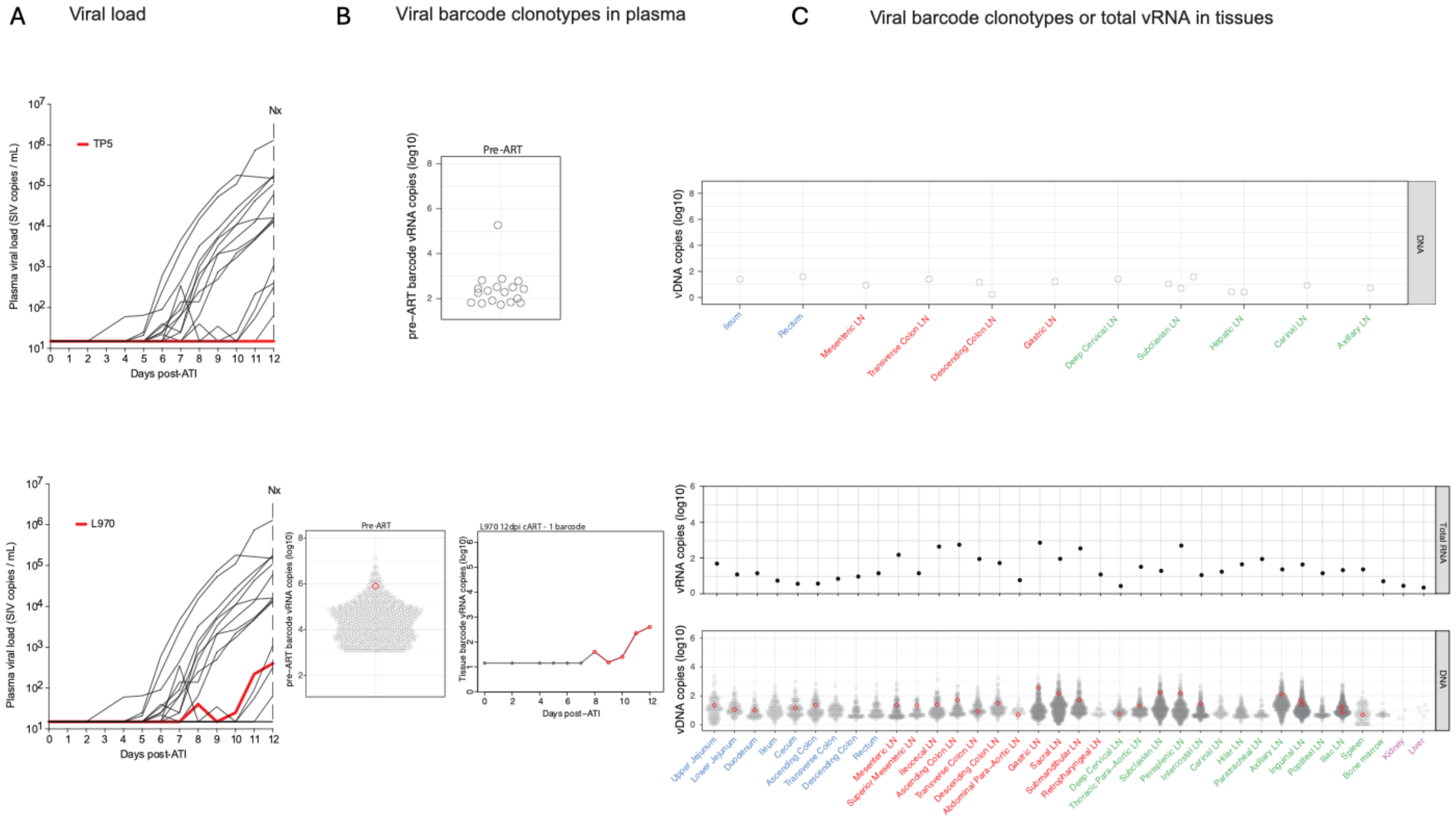

Figure S7.

bioRxiv preprint doi: https://doi.org/10.1101/2025.08.30.673277; this version posted September 1, 2025. The copyright holder for this preprint (which was not certified by peer review) is the author/funder, who has granted bioRxiv a license to display the preprint in perpetuity. It is made available under aCC-BY 4.0 International license.

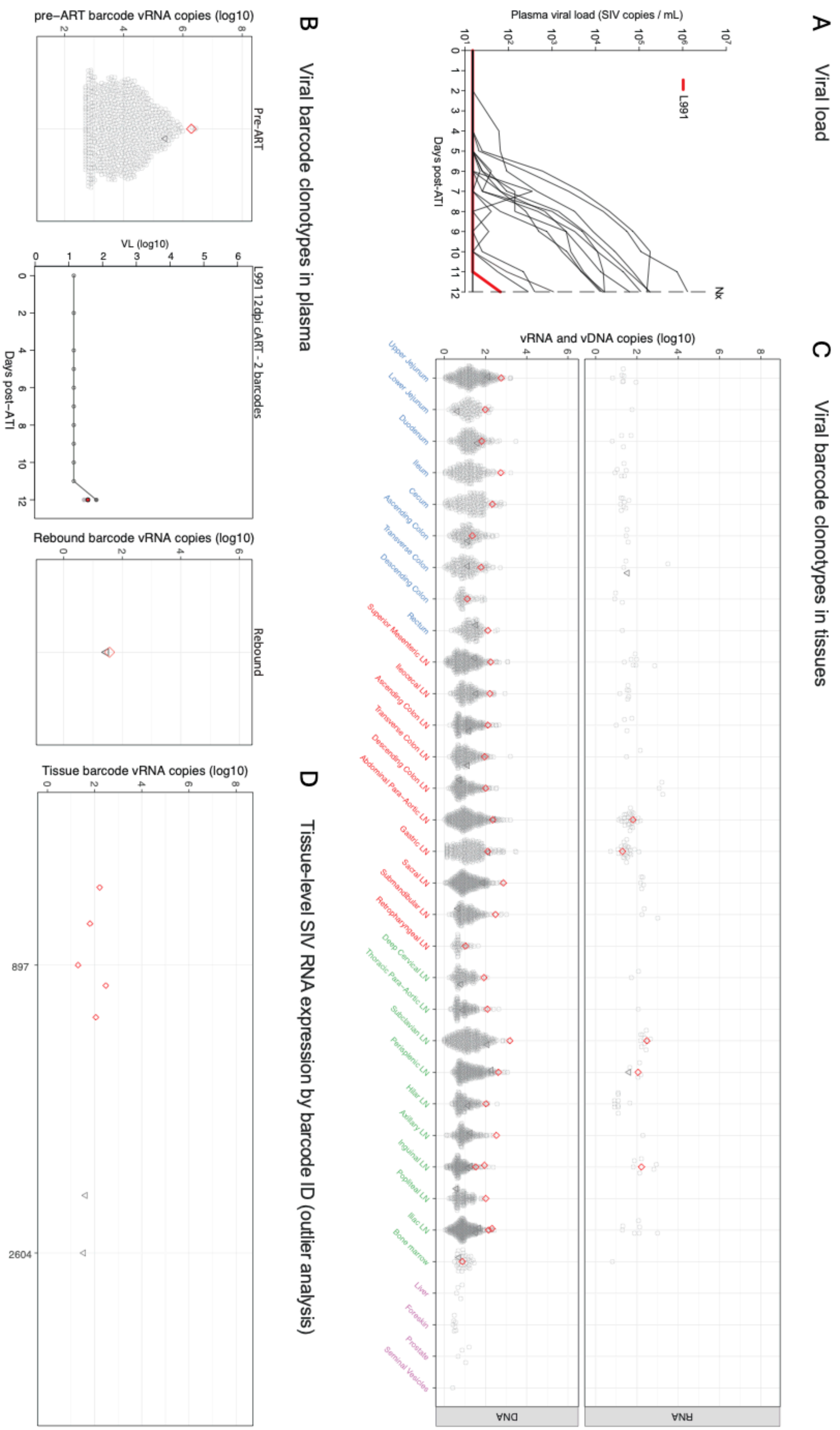

Figure S8.

bioRxiv preprint doi: https://doi.org/10.1101/2025.08.30.673277; this version posted September 1, 2025. The copyright holder for this preprint (which was not certified by peer review) is the author/funder, who has granted bioRxiv a license to display the preprint in perpetuity. It is made available under aCC-BY 4.0 International license.

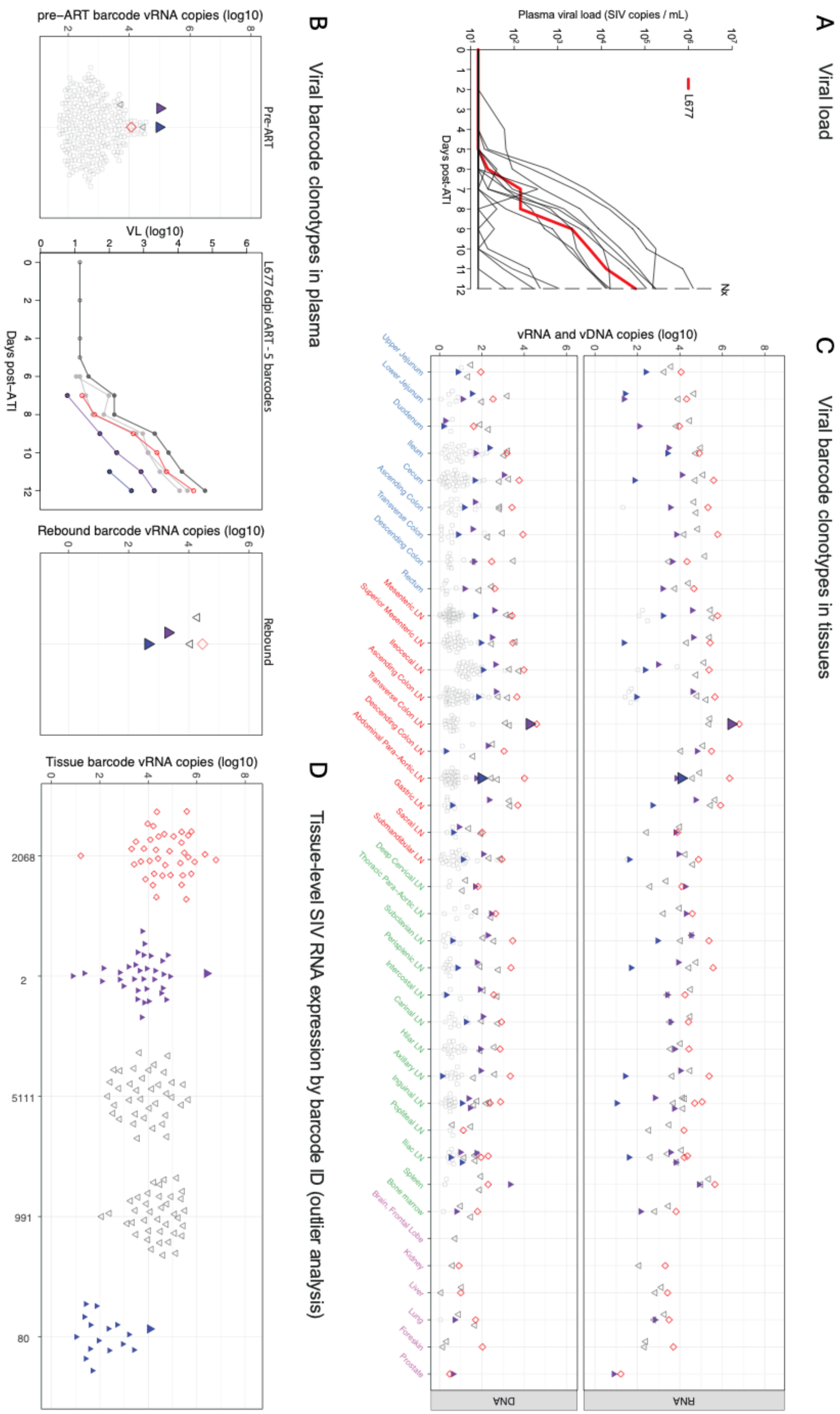

Figure S9.

bioRxiv preprint doi: https://doi.org/10.1101/2025.08.30.673277; this version posted September 1, 2025. The copyright holder for this preprint (which was not certified by peer review) is the author/funder, who has granted bioRxiv a license to display the preprint in perpetuity. It is made available under aCC-BY 4.0 International license.

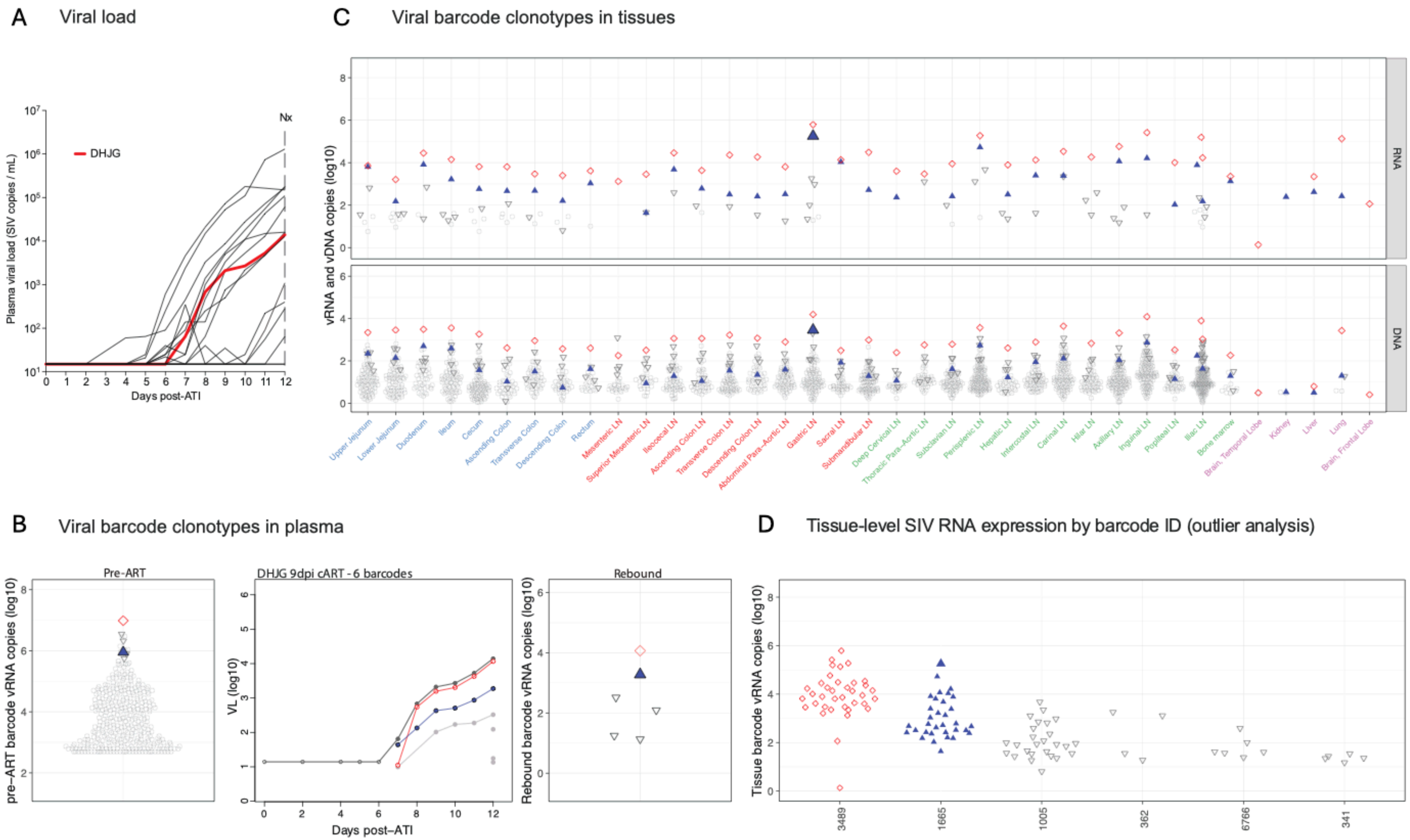

Figure S10.

bioRxiv preprint doi: https://doi.org/10.1101/2025.08.30.673277; this version posted September 1, 2025. The copyright holder for this preprint (which was not certified by peer review) is the author/funder, who has granted bioRxiv a license to display the preprint in perpetuity. It is made available under aCC-BY 4.0 International license.

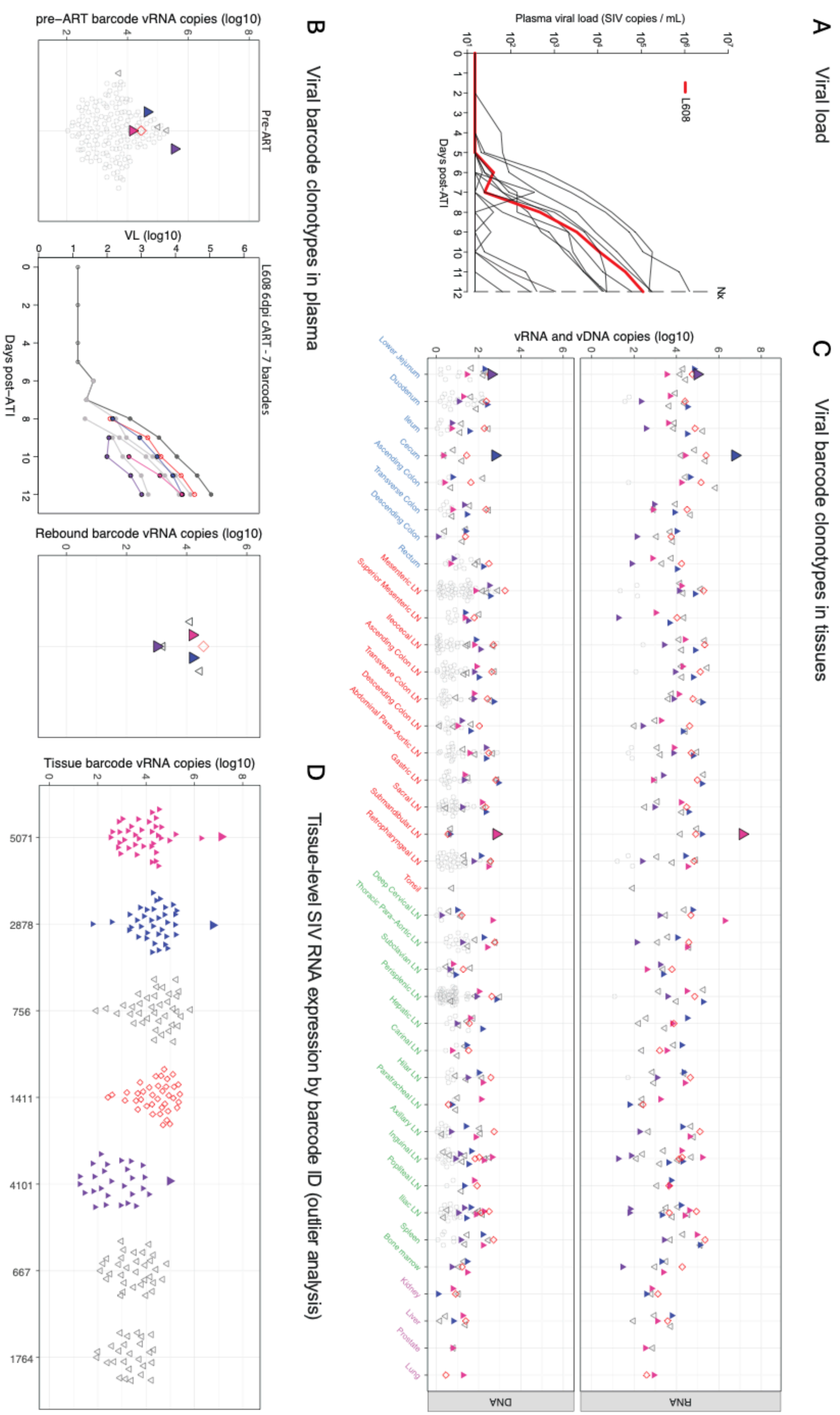

Figure S11.

bioRxiv preprint doi: https://doi.org/10.1101/2025.08.30.673277; this version posted September 1, 2025. The copyright holder for this preprint (which was not certified by peer review) is the author/funder, who has granted bioRxiv a license to display the preprint in perpetuity. It is made available under aCC-BY 4.0 International license.

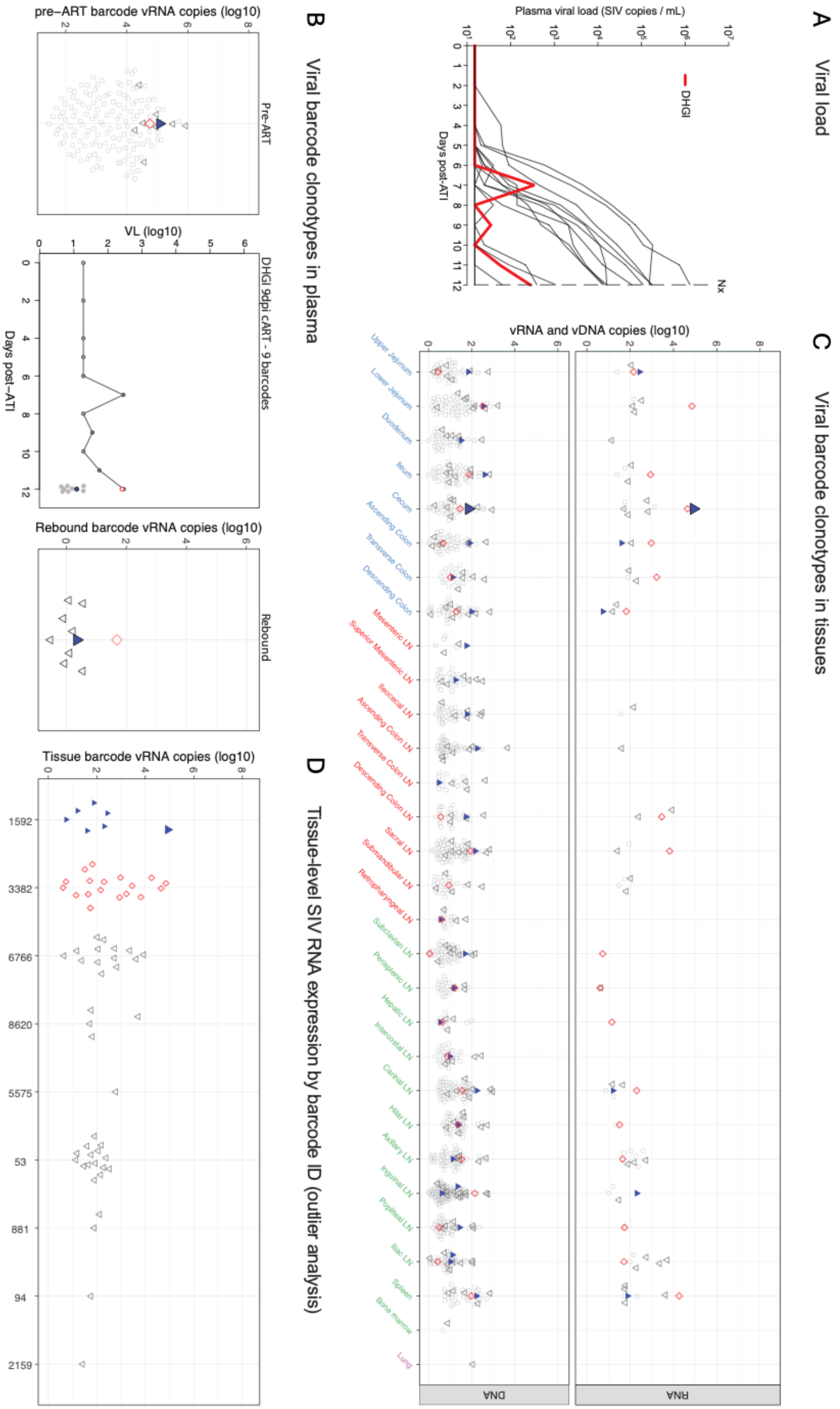

Figure S12.

bioRxiv preprint doi: https://doi.org/10.1101/2025.08.30.673277; this version posted September 1, 2025. The copyright holder for this preprint (which was not certified by peer review) is the author/funder, who has granted bioRxiv a license to display the preprint in perpetuity. It is made available under aCC-BY 4.0 International license.

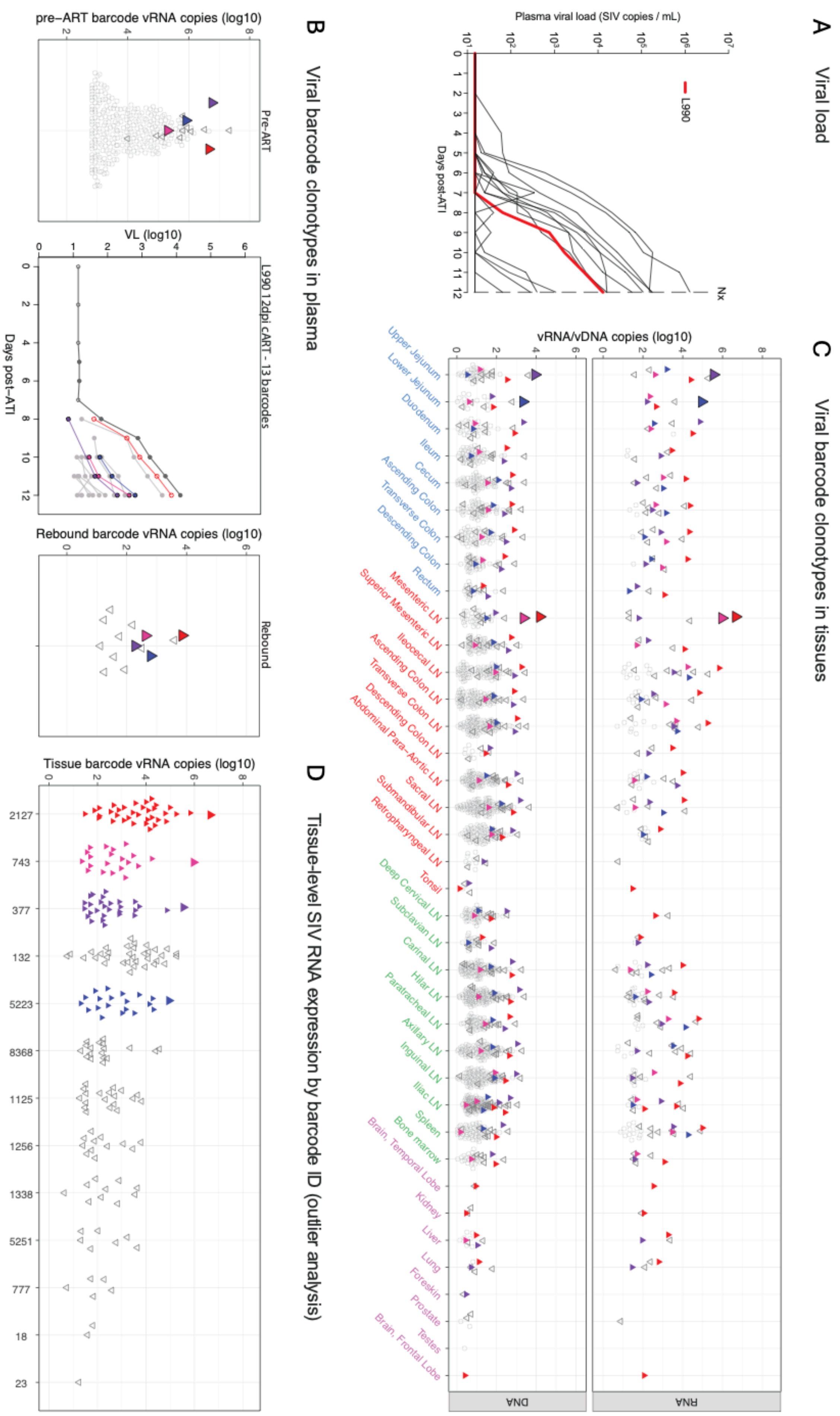

Figure S13.

bioRxiv preprint doi: https://doi.org/10.1101/2025.08.30.673277; this version posted September 1, 2025. The copyright holder for this preprint (which was not certified by peer review) is the author/funder, who has granted bioRxiv a license to display the preprint in perpetuity. It is made available under aCC-BY 4.0 International license.

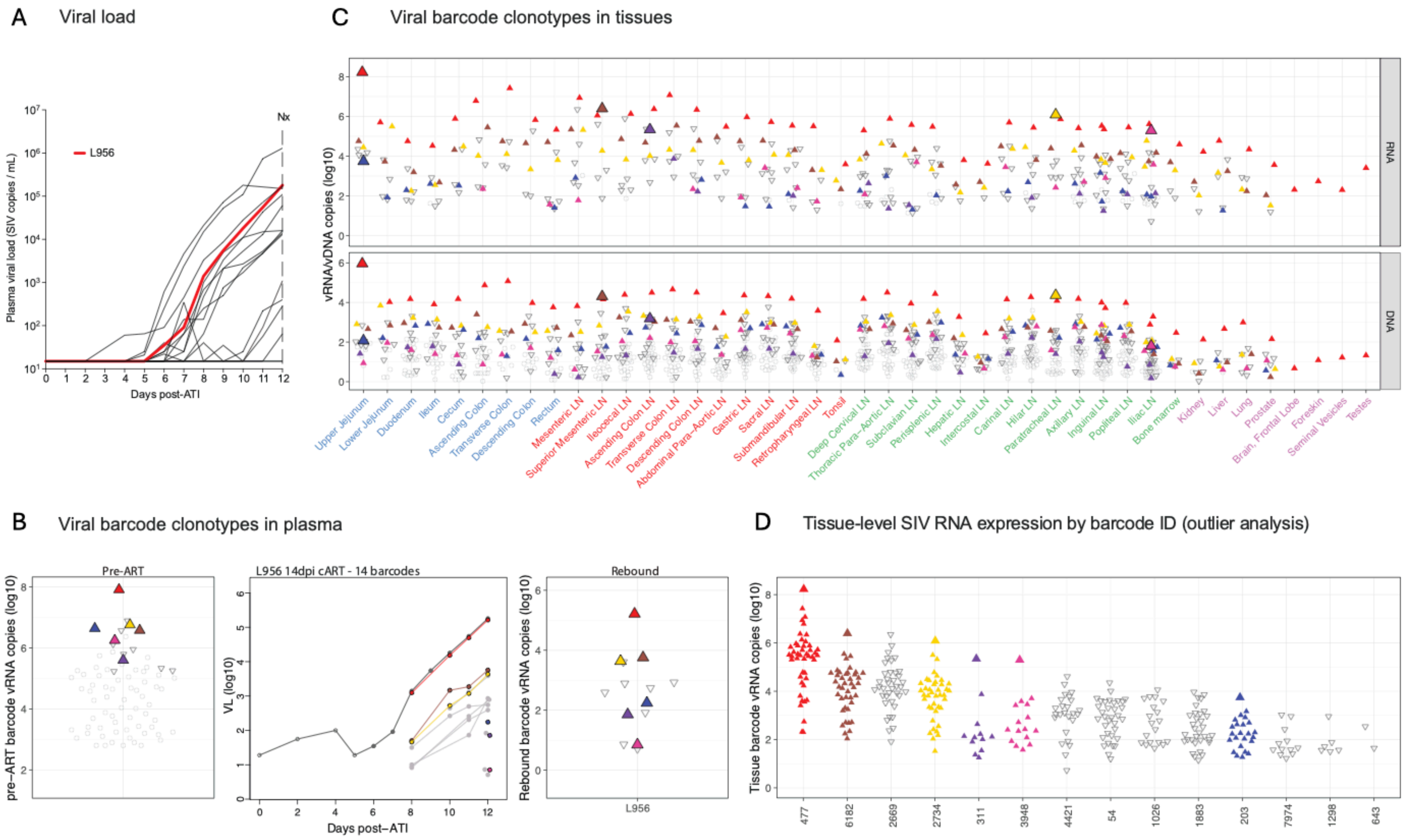

Figure S14.

bioRxiv preprint doi: https://doi.org/10.1101/2025.08.30.673277; this version posted September 1, 2025. The copyright holder for this preprint (which was not certified by peer review) is the author/funder, who has granted bioRxiv a license to display the preprint in perpetuity. It is made available under aCC-BY 4.0 International license.

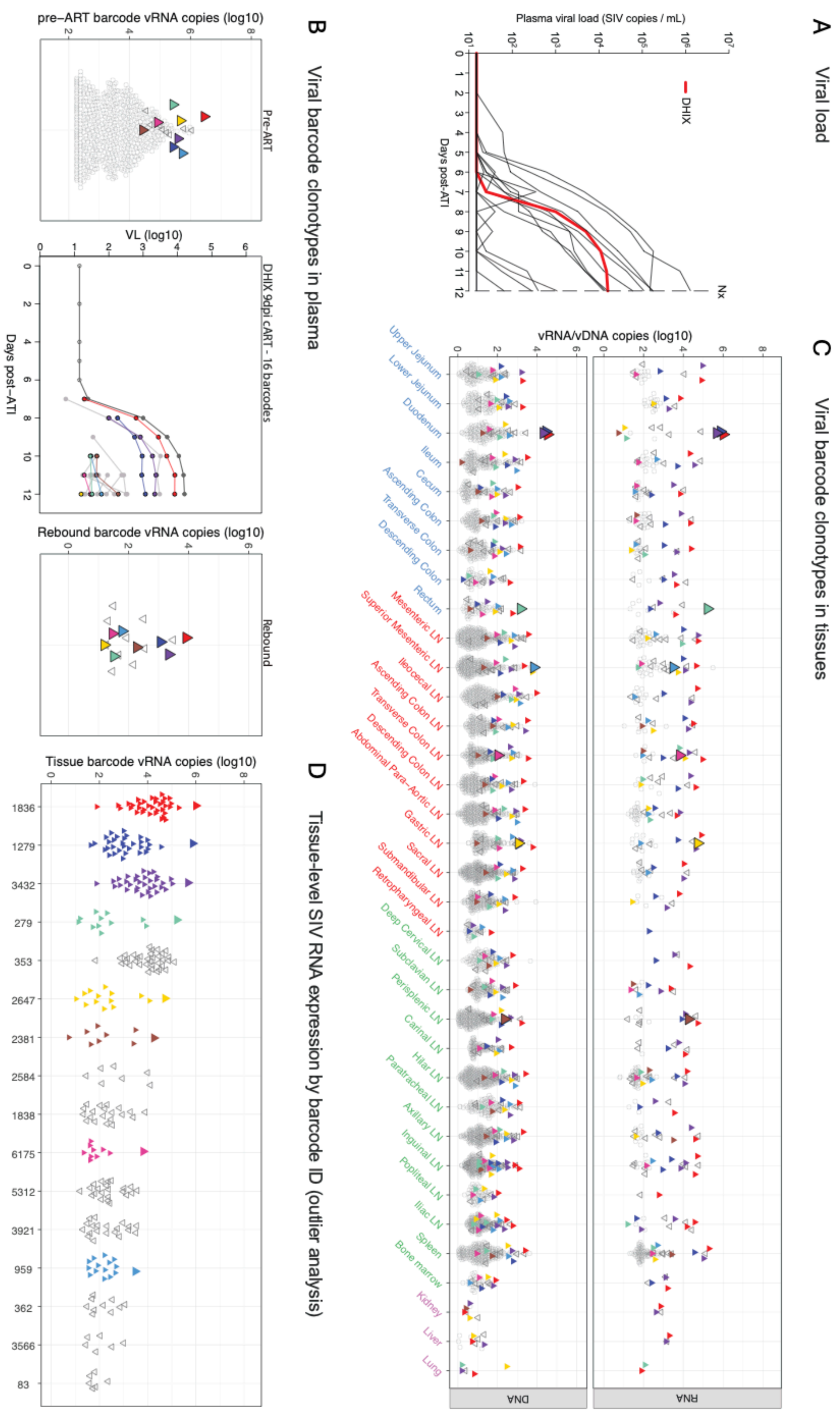

Figure S15.

bioRxiv preprint doi: https://doi.org/10.1101/2025.08.30.673277; this version posted September 1, 2025. The copyright holder for this preprint (which was not certified by peer review) is the author/funder, who has granted bioRxiv a license to display the preprint in perpetuity. It is made available under aCC-BY 4.0 International license.

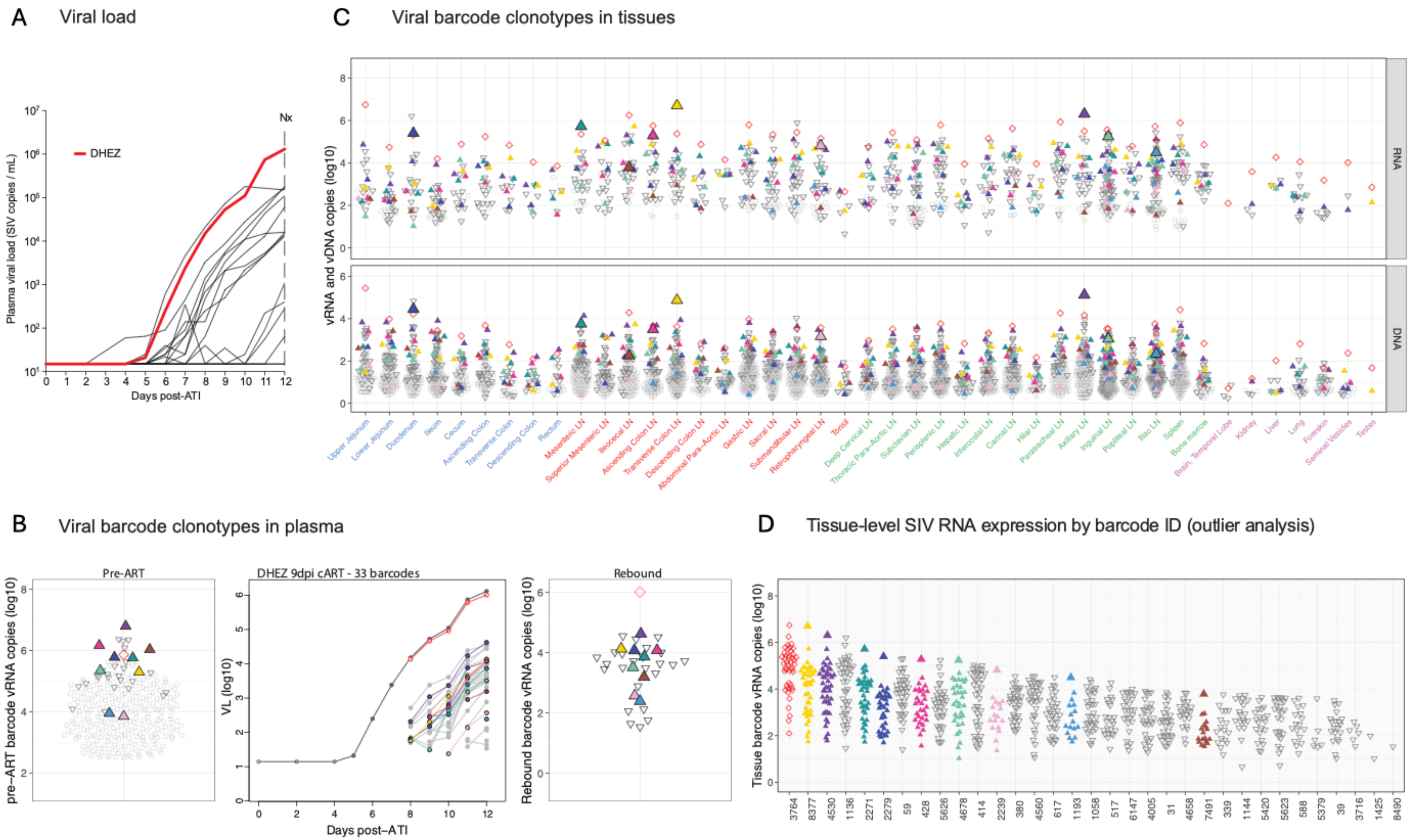

Figure S16.

bioRxiv preprint doi: https://doi.org/10.1101/2025.08.30.673277; this version posted September 1, 2025. The copyright holder for this preprint (which was not certified by peer review) is the author/funder, who has granted bioRxiv a license to display the preprint in perpetuity. It is made available under aCC-BY 4.0 International license.

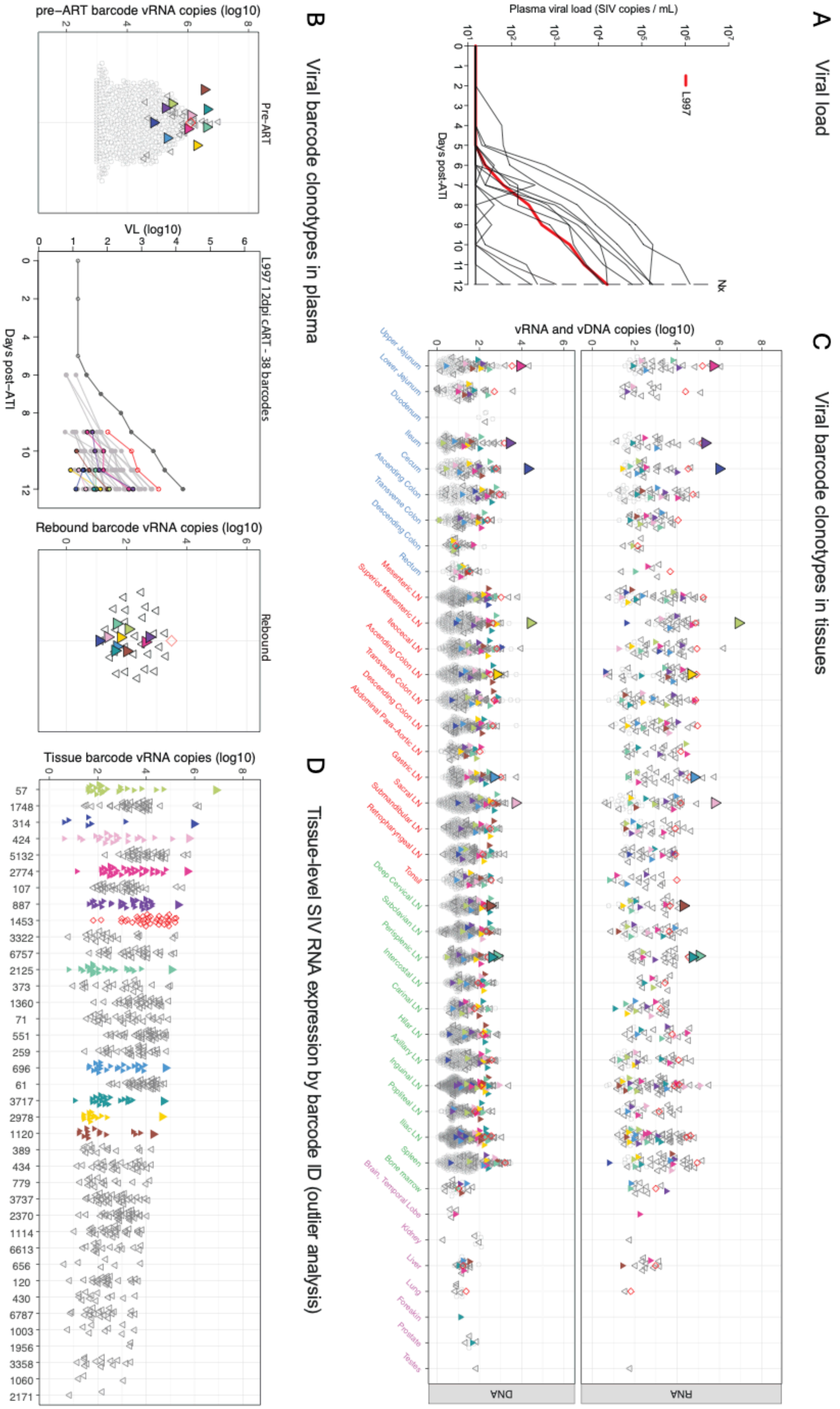

Figure S17.

bioRxiv preprint doi: https://doi.org/10.1101/2025.08.30.673277; this version posted September 1, 2025. The copyright holder for this preprint (which was not certified by peer review) is the author/funder, who has granted bioRxiv a license to display the preprint in perpetuity. It is made available under aCC-BY 4.0 International license.

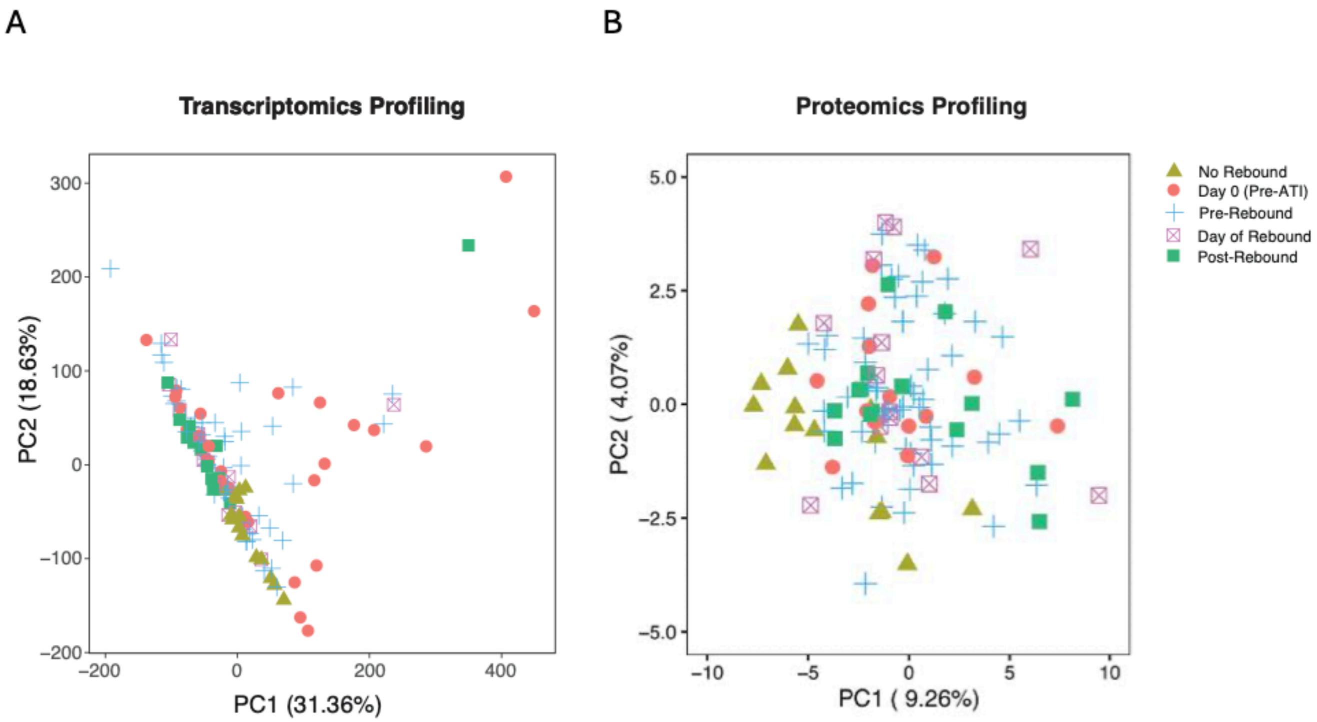

Figure S18.

bioRxiv preprint doi: https://doi.org/10.1101/2025.08.30.673277; this version posted September 1, 2025. The copyright holder for this preprint (which was not certified by peer review) is the author/funder, who has granted bioRxiv a license to display the preprint in perpetuity. It is made available under aCC-BY 4.0 International license.

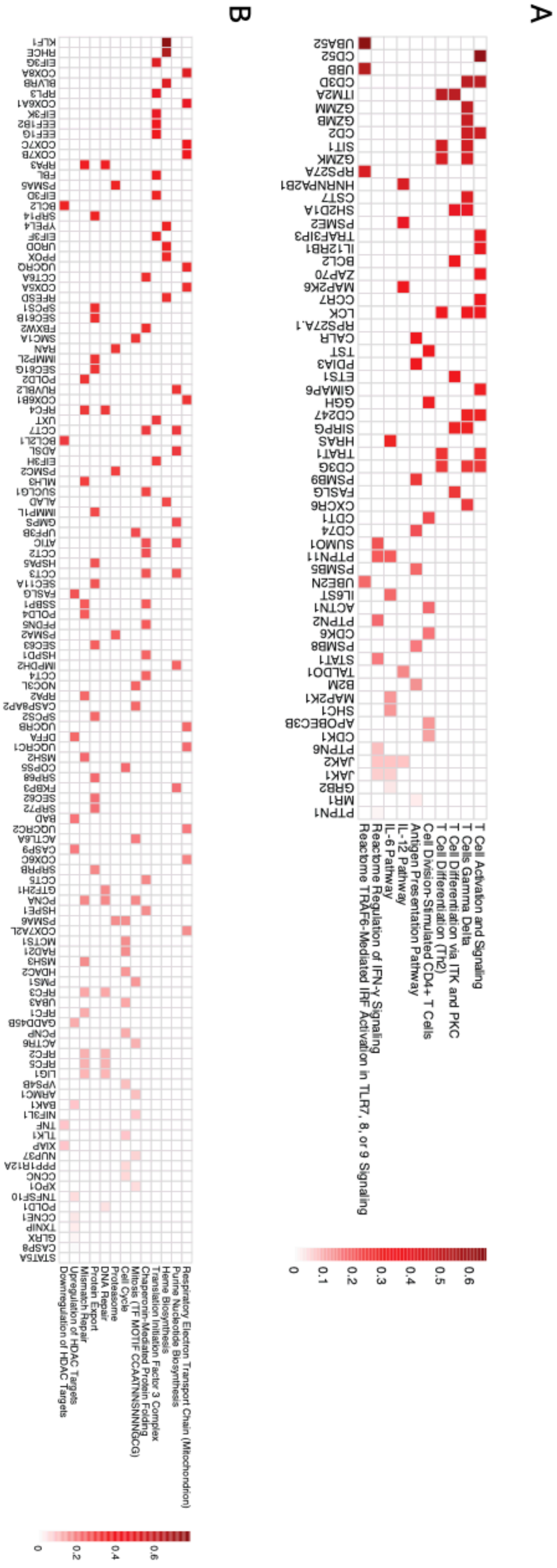

Figure S19.

bioRxiv preprint doi: https://doi.org/10.1101/2025.08.30.673277; this version posted September 1, 2025. The copyright holder for this preprint (which was not certified by peer review) is the author/funder, who has granted bioRxiv a license to display the preprint in perpetuity. It is made available under aCC-BY 4.0 International license.

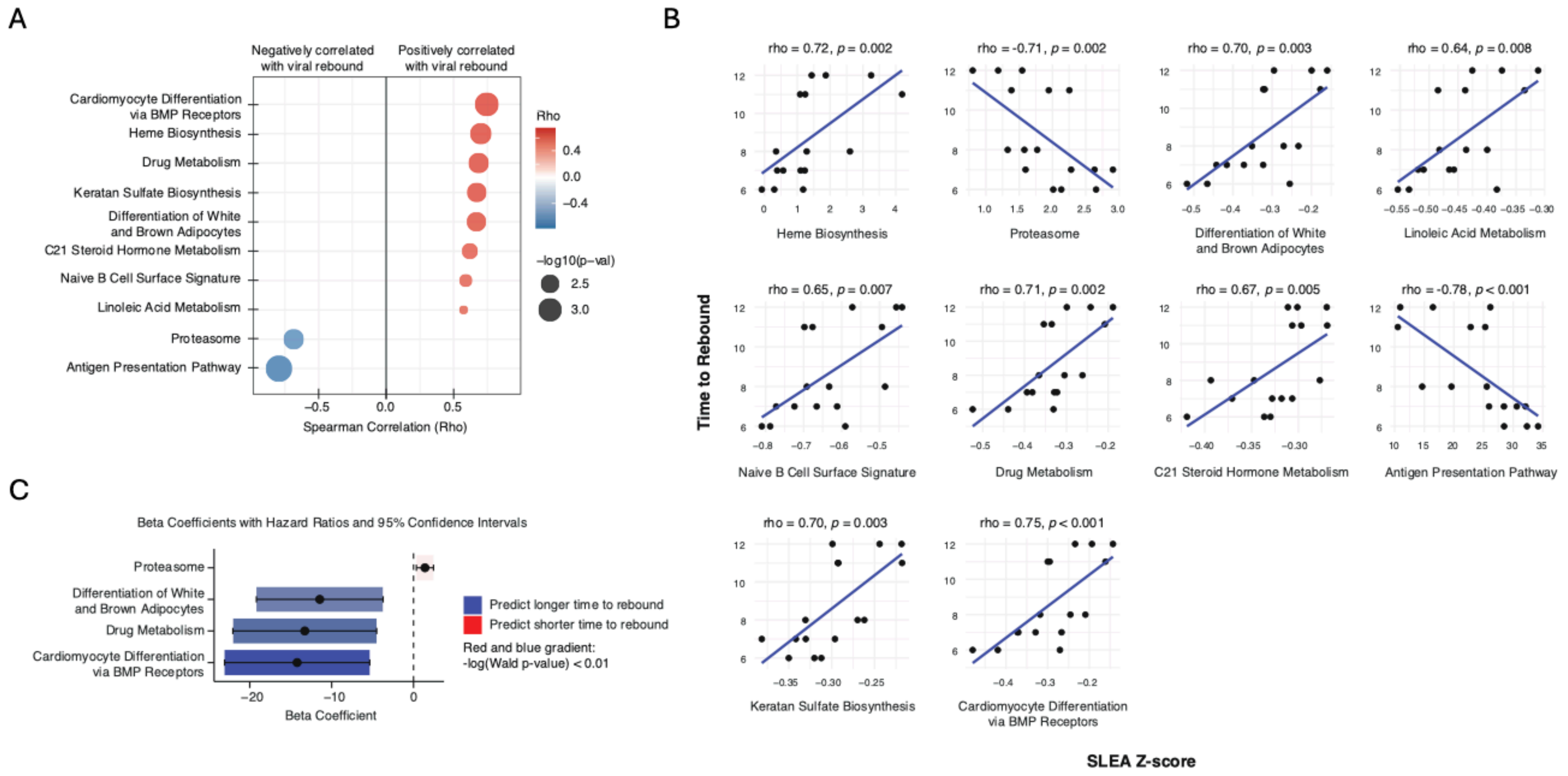

Figure S20.

bioRxiv preprint doi: https://doi.org/10.1101/2025.08.30.673277; this version posted September 1, 2025. The copyright holder for this preprint (which was not certified by peer review) is the author/funder, who has granted bioRxiv a license to display the preprint in perpetuity. It is made available under aCC-BY 4.0 International license.

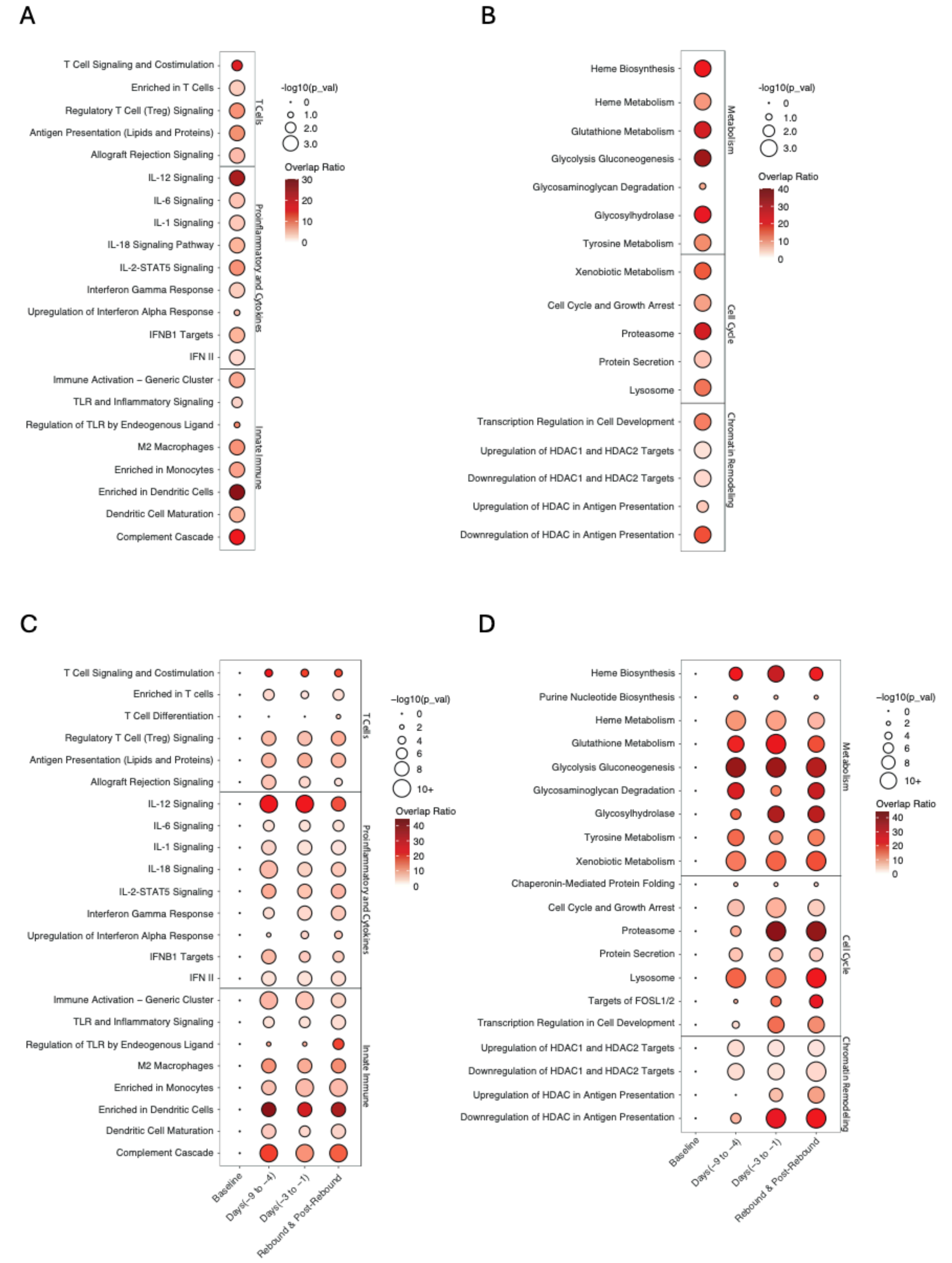

Figure S21.

bioRxiv preprint doi: https://doi.org/10.1101/2025.08.30.673277; this version posted September 1, 2025. The copyright holder for this preprint (which was not certified by peer review) is the author/funder, who has granted bioRxiv a license to display the preprint in perpetuity. It is made available under aCC-BY 4.0 International license.

ART Group	Animal ID	Plasma Viral Load at Necropsy (SIV copies/mL)	Rebound Growth Rate (log RNA/day)	Number of Barcodes	Reactivation Rate (events/day)
Day 6	J639	1,100	2.03	1	0.12
	L604	180,000	2.22	3	0.49
	L608	110,000	1.58	7	0.84
	L677	61,000	1.29	5	0.6
	TP5	< 50	N/A	N/A	N/A
Day 9	DHGI	575	1.7	11	0.59
	DHEZ	1,300,000	2.05	34	5.67
	DHIX	16,000	2.65	8	1.38
	DHJG	14,000	1.74	6	0.6
	DHHN	< 50	N/A	N/A	N/A
Day 12	L681	170,000	1.8	22	2.65
	L956	180,000	1.79	14	1.29
	L970	400	1.39	1	0.13
	L990	13,000	1.63	14	1.53
	L991	65	1.54	2	0.23
	L997	16,000	1.1	38	4.23

Table S1.

bioRxiv preprint doi: https://doi.org/10.1101/2025.08.30.673277; this version posted September 1, 2025. The copyright holder for this preprint (which was not certified by peer review) is the author/funder, who has granted bioRxiv a license to display the preprint in perpetuity. It is made available under aCC-BY 4.0 International license.

Animal ID	Sex	Age at Study Start (Years)	Day of ART Initiation	Number of Tissues Collected at Necropsy
**DHEZ**	Male	2.5	9	57
**DHGI**	Male	2.7	9	51
**DHHN**	Male	2.7	9	85
**DHIX**	Male	2.4	9	56
**DHJG**	Male	2.7	9	81
**J639**	Male	4.3	6	60
**TP5**	Female	4.5	6	54
**L604**	Male	2.5	6	62
**L608**	Male	2.5	6	71
**L677**	Male	2.4	6	76
**L681**	Male	2.5	12	52
**L956**	Male	2.4	12	62
**L970**	Male	2.4	12	73
**L990**	Male	2.4	12	55
**L991**	Male	2.5	12	62
**L997**	Male	2.4	12	71

Table S2.

bioRxiv preprint doi: https://doi.org/10.1101/2025.08.30.673277; this version posted September 1, 2025. The copyright holder for this preprint (which was not certified by peer review) is the author/funder, who has granted bioRxiv a license to display the preprint in perpetuity. It is made available under aCC-BY 4.0 International license.

A

Effect of pre-ART VL (log10) on probability of rebound

	Null model			Pre-ART model		
Predictors	Odds Ratios	CI	p	Odds Ratios	CI	p
(Intercept)	0.02	0.01 – 0.06	<0.001	0	0.00 – 0.00	<0.001
Pre-ART barcode VL (log10)				14.59	10.48 – 20.30	<0.001
Random Effects						
σ^2	3.29			3.29		
τ_{00}	2.43 animal			4.16 animal		
ICC	0.43			0.56		
N	16 animal			16 animal		
Observations	4893			4893		
Marginal R^2 / Conditional R^2	0.000 / 0.425			0.500 / 0.779		
AICc	1318			769		

B

Effect of total tissue barcode vDNA and vRNA on rebound barcode pVL

	Rebound pVL vs tissue vDNA			Rebound pVL vs tissue vRNA		
Predictors	Estimates	CI	p	Estimates	CI	p
(Intercept)	-0.38	- 1.07 – 0.31	0.277	-0.06	-0.56 – 0.44	0.819
total tissue vDNA (log10)	0.83	0.69 – 0.96	<0.001			
total tissue vRNA (log10)				0.58	0.51 – 0.65	<0.001
Random Effects						
σ^2	0.41			0.31		
τ_{00}	0.67 animal			0.42 animal		
ICC	0.62			0.58		
N	14 animal			14 animal		
Observations	167			167		
Marginal R^2 / Conditional R^2	0.259 / 0.718			0.438 / 0.763		
AICc	373.678			322.856		

C

Effect of tissue group on probability of rebound

	Null Model			Tissue type model		
Predictors	Odds Ratios	CI	p	Odds Ratios	CI	p
(Intercept)	0.13	0.08 – 0.21	<0.001	0.06	0.03 – 0.13	<0.001
Non-GI LNs				1		
GI tract				2.57	1.12 – 5.87	0.026
Gut mucosa draining LNs				3.07	1.42 – 6.64	0.004
Random Effects						
σ^2	3.29			3.29		
τ_{00}	0.27 id			0.29 id		
ICC	0.08			0.08		
N	11 id			11 id		
Observations	380			380		
Marginal R^2 / Conditional R^2	0.000 / 0.077			0.070 / 0.146		
AICc	289.104			283.679		

Null model vs tissue group model

Df	Chisq	Pr(>Chisq)
2	9.4	0.0087

Table S3.
